# Bioinspired Directional Hydrogel‐Based High‐Performance Flexible Sensor for Multiple Jumping Pattern Detection in Athletic Training

**DOI:** 10.1002/advs.202515261

**Published:** 2025-10-20

**Authors:** Hanqi Wang, Sen Wang, Yirong Jiang, Zhehao Han, Da Lin, Qinglu Luo, Tianqi Fu, Hanyi Zhang, Deshuai Yu, Jia Yi, Yan Hu, Youhui Lin

**Affiliations:** ^1^ National Institute for Data Science in Health and Medicine Xiamen University Xiamen 361102 P. R. China; ^2^ Department of Physics Research Institute for Biomimetics and Soft Matter Fujian Provincial Key Laboratory for Soft Functional Materials Research Xiamen University Xiamen 361005 P. R. China; ^3^ The Department of Gynecology The First Affiliated Hospital of Wenzhou Medical University Wenzhou Zhejiang 325000 P. R. China; ^4^ Department of Rehabilitation the Tenth Affiliated Hospital of Southern Medical University Dongguan People's Hospital Dongguan 523000 P. R. China; ^5^ Dongguan Experimental Centre for Sports Rehabilitation Research Dongguan 523000 P. R. China; ^6^ Xiamen University Affiliated Keji High School Xiamen 361102 P. R. China

**Keywords:** anisotropic structure, bioinspired materials, conductive hydrogel, flexible sensor, moisture retention

## Abstract

Conductive hydrogels have garnered significant attention as ideal materials for flexible wearable sensors due to their conductivity, flexibility, adaptability, and biocompatibility. However, traditional conductive hydrogels frequently exhibit poor moisture retention and suboptimal mechanical properties, which greatly limit their practical usability. Inspired by the anisotropic structure of biological tissues and the natural moisturizing factors in skin, a novel bioinspired directional hydrogel (BDH) system is presented, using polyvinyl alcohol as the matrix, incorporating polydopamine‐modified carbon nanotubes and PEDOT‐PSS as conductive materials, and sodium pyrrolidone carboxylic acid for moisture retention. The precursor solution containing disordered polymer chains undergoes flow‐induced alignment, followed by strong aggregation and crystallization driven by a kosmotropic salt solution. This dual‐stage process ultimately yields the BDH with pronounced structural anisotropy, characterized by tightly packed, aligned polymer domains. The obtained hydrogel exhibits excellent mechanical strength, damage tolerance, good conductivity, and moisture retention, making it suitable as a flexible sensor for high‐load stress conditions. When combined with machine learning algorithms, BDHs enable accurate motion tracking and intent recognition, showing promising applications in motor training and ability assessment. This efficient, energy‐saving fabrication method offers a promising strategy for developing bioinspired structural hydrogels, facilitating their practical use in human‐machine interactions.

## Graphical abstract

This study develops a bioinspired directional hydrogel (BDH) via flow‐induced alignment of polyvinyl alcohol and dispersed carbon nanotubes, enhanced by the immersion of natural moisturizing factors. The BDH exhibits superior mechanical strength, conductivity, and moisture retention, enabling high‐performance flexible sensors for precise motion tracking in athletic training when integrated with machine learning algorithms.

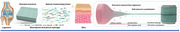

## 1 Introduction

The development of soft sensors has gained significant attention in recent years, particularly in bridging the gap between humans and machines.^[^ ^1^, ^2^, ^3^ ^]^ Conductive hydrogels,^[^ ^4^, ^5^, ^6^ ^]^ as key materials in soft sensors, exhibit unique properties including flexibility, high water content, and biocompatibility, making them ideal for wearable devices and human‐machine interfaces. Typically, conductive hydrogels are engineered through the integration of conductive polymers,^[^ ^7^, ^8^ ^]^ carbon‐based materials,^[^ ^9^ ^]^ metal‐based materials,^[^ ^10^ ^]^ or ionic side groups and salts into flexible 3D hydrogel frameworks.^[^ ^11^, ^12^, ^13^ ^]^ However, highly conductive hydrogels often become brittle due to the high concentration of rigid conductive materials, while materials with high hydration levels are generally softer and less conductive.^[^ ^14^, ^15^ ^]^ Additionally, hydrogels are usually susceptible to dehydration, resulting in unstable performance. Moreover, most of the existing conductive hydrogels are synthesized via in situ polymerization, ion chelation, or physical blending, which typically lead to isotropic and disordered internal structures.^[^ ^16^, ^17^, ^18^, ^19^, ^20^ ^]^ Such architectures lack resistance to mechanical cracking and fatigue, severely limiting their performance in dynamic and load‐bearing environments. Therefore, there is an urgent need for advanced hydrogels that can achieve a synergistic balance among mechanical robustness, electrical conductivity, and water‐retention capacity, while also offering ease of fabrication and adaptability for diverse applications.^[^ ^21^ ^]^

To overcome these limitations, researchers have increasingly drawn inspiration from biological tissues by introducing anisotropic structures into hydrogels.^[^ ^22^, ^23^, ^24^ ^]^ In natural systems, regular dense connective tissues (e.g., skin, tendons, and ligaments) rely on aligned collagen fibers to endure directional forces and provide structural resilience.^[^ ^25^ ^]^ Inspired by this architecture, anisotropic hydrogels have been developed to enhance mechanical performance while preserving flexibility. Notably, introducing anisotropic structures can also improve electrical conductivity by promoting directional charge transport, thereby coupling mechanical and electrical performance.^[^ ^26^ ^]^ Among various fabrication techniques, extrusion‐induced alignment has emerged as an effective strategy for constructing directional polymer networks. However, when combined with salt‐based coagulation baths, this method often accelerates water loss, thereby compromising the hydrogel's long‐term hydration. To address this issue, moisture‐retaining components, such as natural moisturizing factors (NMFs), have been introduced into the hydrogel network to improve water retention. Therefore, the seamless integration of directional alignment and hydration‐preserving strategies is critical for developing next‐generation conductive hydrogels with a balanced combination of mechanical robustness, moisture stability, and adaptability for wearable electronics and human‐machine interfaces.^[^ ^27^, ^28^ ^]^

**Scheme 1:**
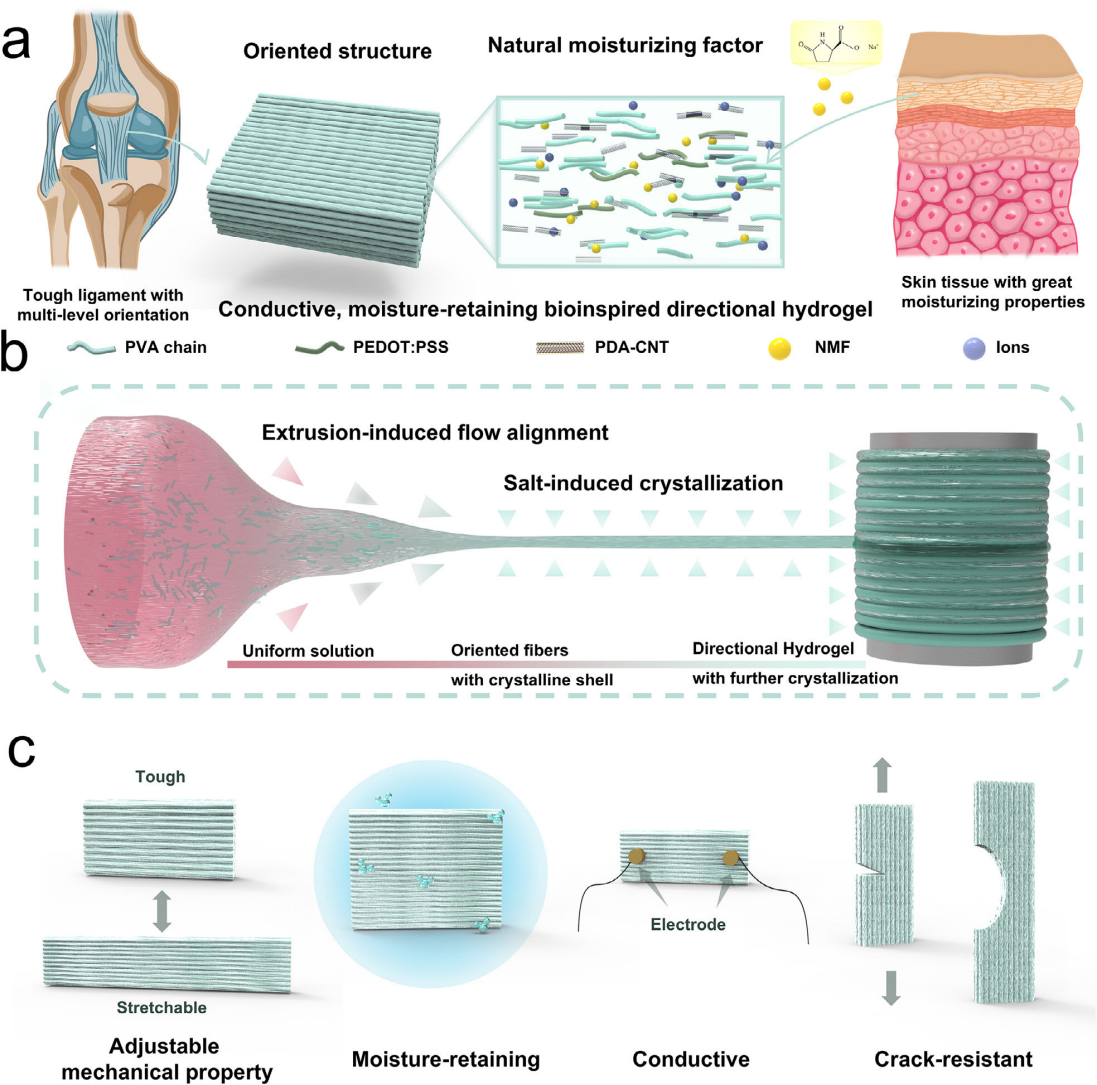
Schematic illustration of the bioinspired design, fabrication process, and multifunctional properties of BDH. a) Bioinspired design of BDH based on directional ligament structures and natural moisturizing factors. b) Fabrication process of BDH involving fiber alignment and directional hydrogel formation. c) Multifunctional properties of BDH, including flexibility, toughness, moisture retention, and crack resistance.

Drawing inspiration from the hierarchical architecture of connective tissues and the hydration function of NMFs in the skin, we present an energy‐efficient and time‐saving strategy for fabricating an innovative bioinspired directional hydrogel (BDH) with tunable mechanical properties and exceptional moisture retention, designed as a high‐performance sensing platform for athletic ability assessment (**Scheme** **1**). This design specifically addresses the limitations of existing flexible sensors in recognizing complex motion patterns, where conventional isotropic hydrogels often lack directional sensitivity and sufficient mechanical robustness. These BDHs, characterized by their precisely ordered multiscale structures, exhibit superior mechanical strength and enhanced crack resistance compared to conventional isotropic hydrogels. Additionally, they demonstrate high electrical conductivity and outstanding moisture retention. Specifically, BDHs achieve a tensile strength of 10.12 ± 0.49 MPa and an electrical conductivity of 2.13 ± 0.16 S/m. The synergy of these properties makes BDHs highly promising for next‐generation stretchable, high‐stress sensor devices capable of monitoring joint dynamics and predicting motion behavior when integrated with machine learning algorithms. The alignment of polymer chains through flow‐induced stretching represents an energy‐efficient approach to constructing bioinspired hydrogels with complex, hierarchical order. Furthermore, the performance of BDHs can be fine‐tuned by incorporating conductive and moisture‐retaining components, expanding their potential applications in bioelectronics, soft robotics, and wearable sensing technologies.

## 2 Results and Discussion

### 2.1 Design Strategies of Bioinspired Directional Hydrogels

Our bioinspired system recapitulates two essential biological paradigms: i) the multiscale fibrillar alignment seen in connective tissues such as ligaments, and ii) the water‐attracting, retention function of NMFs in skin (Scheme 1a). In our design, polyvinyl alcohol (PVA) and carbon nanotubes (CNTs) serve as the primary components, imparting robust mechanical and electrical properties, respectively.^[^ ^29^, ^30^, ^31^ ^]^ PVA is widely recognized for its excellent biocompatibility and programmable engineering properties, making it an ideal candidate for the fabrication of anisotropic hydrogels.^[^ ^24^, ^31^ ^]^ CNTs, particularly when synergistically combined with poly(3,4‐ethylenedioxythiophene) polystyrene sulfonate (PEDOT:PSS), can act as conductive fillers. To obtain a well‐dispersed conductive ink of CNTs, polydopamine (PDA) is employed as a stabilizer and dispersant to ensure uniform CNT dispersion. Inspired by the moisturizing role of NMFs in the stratum corneum, sodium pyrrolidone carboxylic acid (PCA‐Na) as an important compound of NMFs was selected for its hydrating properties.^[^ ^32^ ^]^ More importantly, PCA‐Na can also function as a kosmotropic salt that promotes aggregation and crystallization of BDHs. To mimic the oriented structure of ligaments, BDHs were fabricated via flow‐induced alignment of the precursor solution, followed by immersion in PCA‐Na, resulting in a naturally multiscale oriented architecture and enhanced mechanical performance (Scheme 1b). Consequently, our rationally engineered BDH demonstrates adjustable mechanical performance, robust electrical conductivity, and excellent moisture retention (Scheme 1c), making it a promising candidate for high‐performance flexible sensors.

### 2.2 Preparation and Characterization of Polydopamine‐modified Carbon Nanotubes

**Figure 1:**
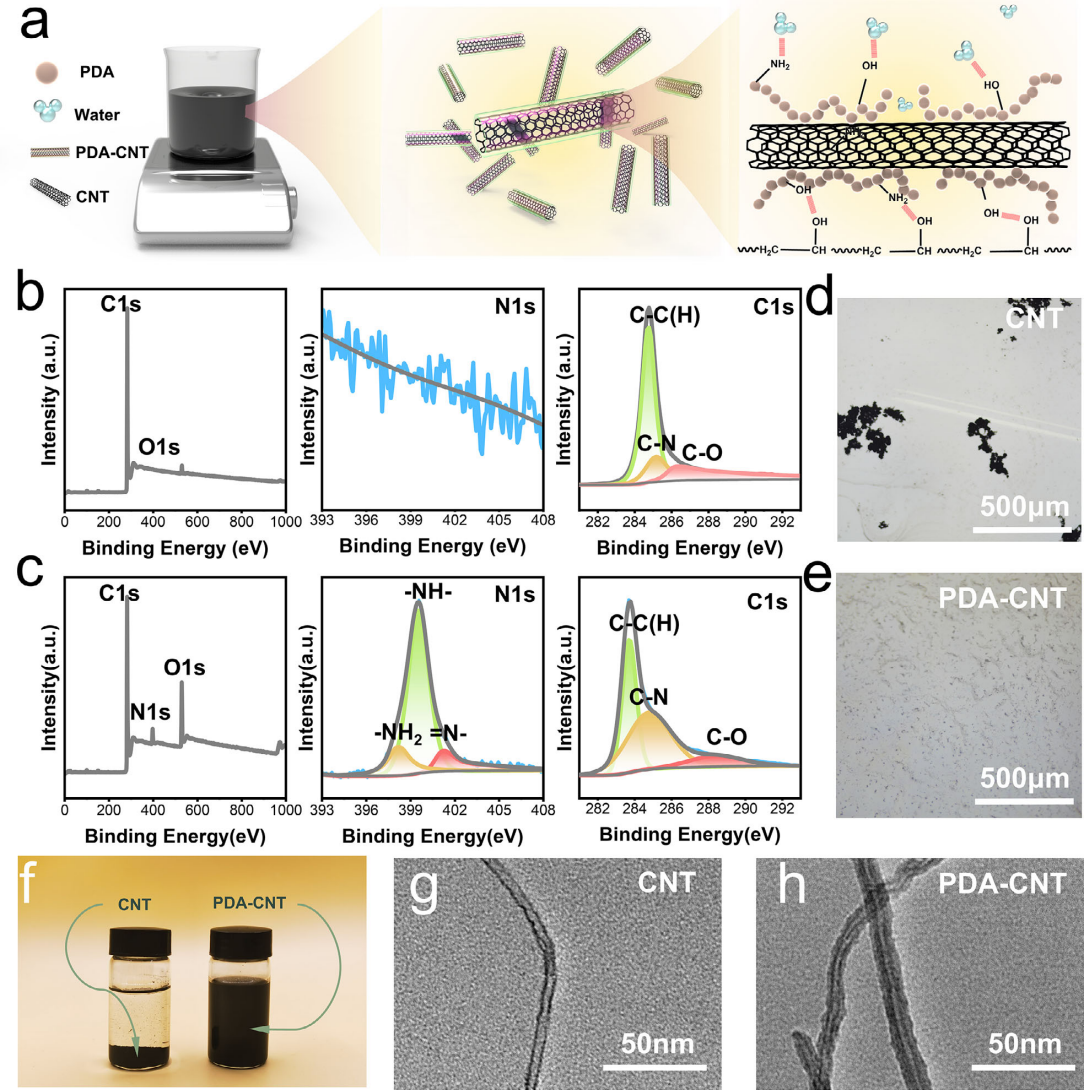
Characterization of PDA‐CNTs. a) Distribution state of PDA‐CNTs in the precursor solution. Full XPS spectrum, N1s spectrum, C1s spectrum of b) CNTs and c) PDA‐CNTs. d) Aggregation of CNTs observed under an optical microscope. e) Dispersion of PDA‐CNTs observed under an optical microscope. f) Typical photographs of CNTs and PDA‐CNTs suspension. g) TEM image of CNTs. h) TEM image of PDA‐CNTs.

Before fabricating well‐ordered BDHs, a stable, well‐dispersed CNT ink was prepared by modifying CNT surfaces with PDA, as schematically illustrated in **Figure** **1**a. Following established protocols, dopamine hydrochloride was introduced into an alkaline aqueous dispersion of CNTs and allowed to react for 24 h. During this period, dopamine underwent spontaneous oxidative polymerization, forming a dark polymer PDA that adheres to CNT surfaces via noncovalent interactions.^[^ ^33^ ^]^ The resulting PDA coating is rich in hydrophilic amino and hydroxyl groups, which not only enhance the compatibility of CNTs with aqueous media but also prevent their aggregation, thereby ensuring a uniform and stable dispersion that forms a homogeneous conductive pathway.^[^ ^34^ ^]^ Furthermore, the PDA layer forms catechol‐hydroxyl interactions with PVA chains, contributing to enhanced energy dissipation during deformation via synergistic noncovalent interactions among PDA‐CNTs, PEDOT:PSS, and PVA.^[^ ^35^ ^]^

To confirm the successful incorporation of polydopamine, X‐ray photoelectron spectroscopy (XPS) was utilized to analyze and compare the surface composition and functional groups of the CNTs before and after modification. The wide‐scan spectra reveal that the unmodified CNTs’ surface consists of C1s peak and O1s peak, while the presence of the PDA layer on the CNTs’ surface is confirmed by the appearance of a new N1s peak at ≈400 eV (Figure 1b,c). Additionally, the N1s spectrum of the PDA‐CNTs can be fitted with three peak components: one at 398.2 eV for ─NH_2_ and another at 401.2 eV for = N‐ groups, which contrasts with the absence of these peaks in the spectrum of the unmodified CNTs.^[^ ^35^ ^]^ Fourier Transform Infrared Spectroscopy (FTIR) has also been utilized to characterize the modification of CNTs (Figure S1, Supporting Information). FTIR analysis showed a more intense peak at 3450 cm^−1^ after modification, which could be attributed to the stretching vibration of the N─H bond. The FTIR result provided direct evidence for the successful attachment of PDA to the CNTs.^[^ ^36^ ^]^ The dispersion of CNTs and PDA‐CNTs in water exhibited a significant difference based on images and absorbance curves (Figure 1d,e; Figures S2 and S3, Supporting Information). The dispersion degree of both modified and unmodified carbon nanotubes was recorded over a 10‐min period. The functionalization of PDA onto CNTs led to significantly enhanced aqueous dispersion stability of PDA‐CNTs. Furthermore, to investigate the adsorption of PDA on the CNTs’ surface, transmission electron microscopy (TEM) was employed to directly observe the combination of PDA and CNTs (Figure 1g,h).^[^ ^37^ ^]^ According to the TEM image, it could be clearly observed that PDA was firmly adsorbed on the CNTs’ surface and formed a uniform coating. Taken together, all our results confirm that PDA has been successfully functionalized on the CNTs, leading to a stable dispersion of PDA‐CNTs in aqueous solution (PC‐ink).

### 2.3 Preparation and Structural Characterization of Bioinspired Directional Hydrogels

**Figure 2:**
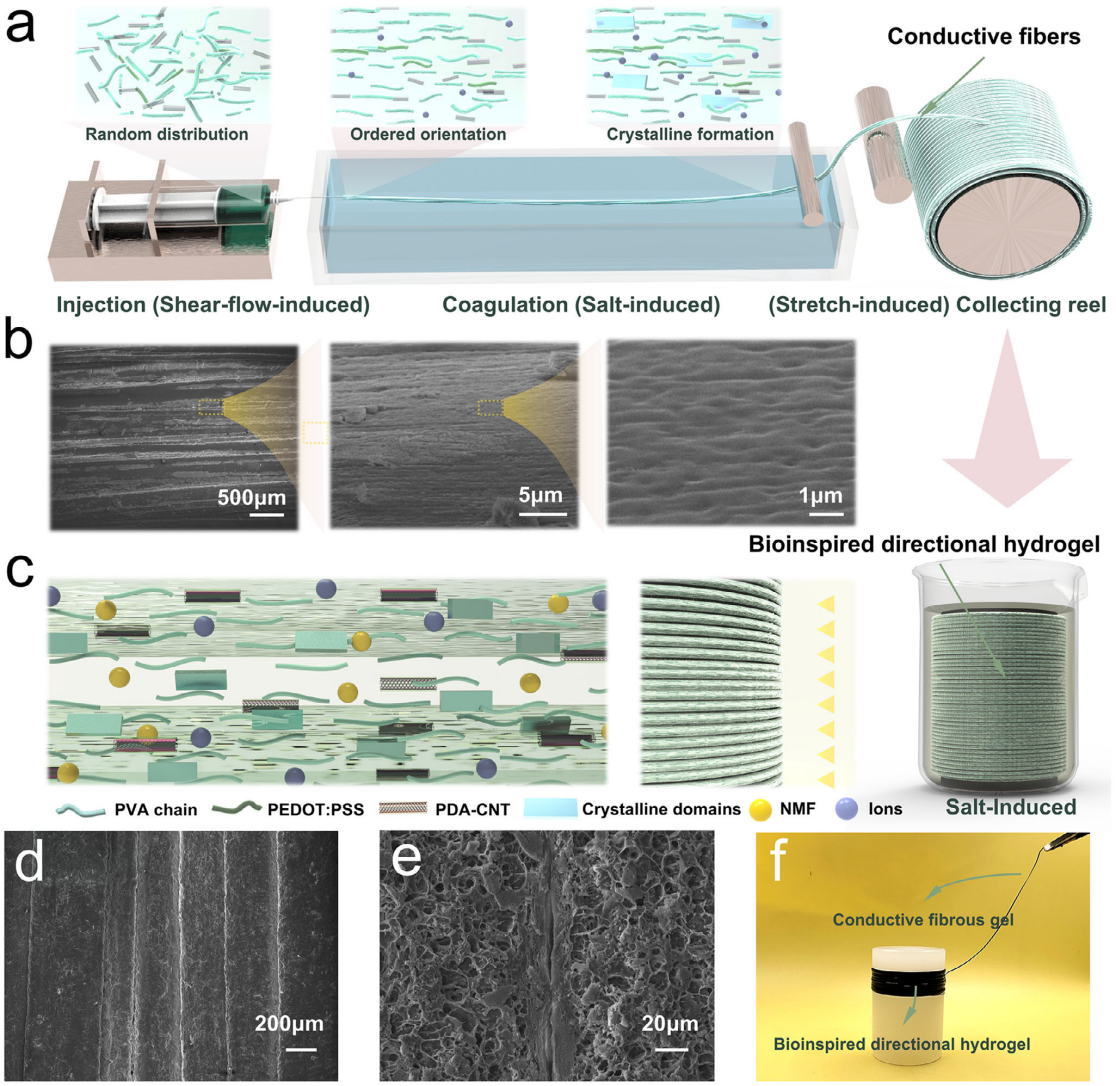
Design strategy for fabricating BDHs. a) Fabrication procedure for well‐ordered nanofibrils using shear‐flow‐induced, salt‐induced, and stretch‐induced alignment. b) Multiscale magnified SEM images showing the preferentially aligned fiber structures. c) Enhancement of nanofibril binding via a salt precipitation process. The inset highlights a magnified view of the localized binding regions. SEM images of adjacent and densely packed fiber filaments, d) on the surface morphology, and e) in the cross‐section. f) Typical photograph of bioinspired directional hydrogels and conductive fibers.

The fabrication process for the bio‐inspired directional hydrogels with anisotropic structure was illustrated schematically in **Figure** **2**. The first and crucial step to manufacture BDHs is the formation of conductive fibers through flow‐induced alignment (Figure 2a), inspired by the wet spinning process used in the textile industry. Specifically, the precursor solution was injected into a solidification bath and rapidly formed fibrous filaments, which would be collected and stretched by a motor‐driven reel. The precursor solution was prepared with PVA, PDA‐CNTs, and PEDOT:PSS, which was then injected into a 40 wt.% ammonium sulfate solution and stretched to form conductive fibers. During this process, the gradient velocity field within the syringe nozzle generated controlled shear forces that induced unidirectional alignment of PVA polymer chains along the flow axis (Figure 2b), thereby establishing well‐defined nanoscale anisotropy. This shear‐induced orientation was subsequently reinforced by the post‐drawing stretching in the coagulation bath. On the macroscopic scale, a highly crystalline shell layer formed rapidly on the surface of the fibers in the coagulation bath, contributing to the overall directionality and crack resistance of the BDHs. The obtained fibers, formed through short‐distance coagulation, were collected onto a motor‐driven reel, after which the entire assembly was immersed in the PCA‐Na solution. During the winding and immersion process, the fibers were firstly stuck together, and subsequently, weak binding forces between fibers were formed, which is mainly due to the crystallization among the amorphous PVA chains in the fiber's outer shell layer (Figure 2c). After soaking for 30 min, the lateral binding forces between the fibers were strengthened, resulting in a piece of hydrogel with ligament‐like structures composed of fiber bundles and interstitial material.

Scanning electron microscopy (SEM) was used to observe the orientation of the BDHs at different scales. As shown in Figure 2b, the BDH consisted of fibrous gels with a tightly stacked alignment. At the nanoscale, the orientation of the nanofiber chains was clearly aligned in the direction of flow, induced by the shear forces during the squeezing and stretching process. These morphological features were in stark contrast to the hydrogels fabricated through freezing‐thawing (FT) or freezing‐soaking (FS) methods (Figures S4–S6, Supporting Information). Meanwhile, SEM analysis revealed the formation of connections between the fibers, which were likely composed of the same components as the fibers, formed through crystallization during extrusion and subsequent solidification (Figure 2d,e; Figure S7, Supporting Information). These results demonstrated that the inter‐fiber connections, formed through crystallization during extrusion and subsequent solidification, significantly enhanced the cohesive properties of BDH, facilitating the formation of a mechanically integrated structure.

**Figure 3:**
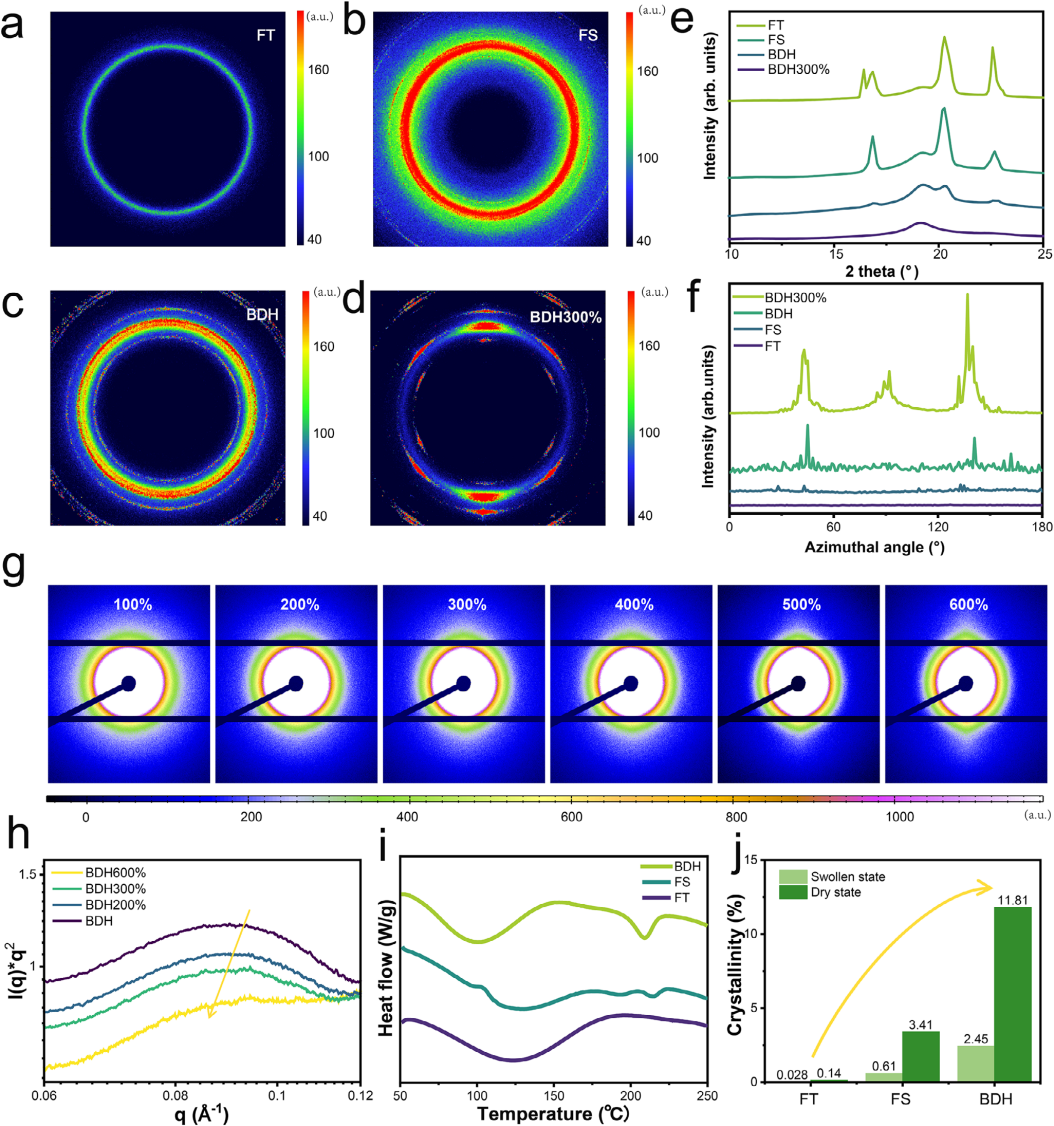
The structural distinction of hydrogels prepared by freezing‐thawing (i.e., FT), freezing‐soaking (i.e., FS), and wet‐spinning (i.e., BDH). 2D WAXS patterns of a) FT hydrogel, b) FS hydrogel, and c) BDH. d) WAXS profiles of FT, FS, BDH, and BDH300% (when the stretch ratio of BDH is 300%). Time evolution of the Cartesian components of the end‐to‐end distance was analyzed under e) shear and f) extensional flow conditions. g) 2D SAXS patterns of BDHs with different stretch ratios. h) SAXS profiles of BDHs with different stretch ratios, i) Corresponding DSC thermographs, and j) crystallinity of the resulting hydrogels in the dry state and swollen state.

To investigate the nanofibrillar structure, wide‐angle X‐ray scattering (WAXS) was also employed to reveal the crystalline domains in the resulting hydrogels (**Figure** **3**a–d). According to the WAXS results, no distinct alignment of crystalline domains was observed in either FT or FS hydrogels. As shown in Figure 3e, BDH displays a sharp diffraction peak at 2θ = 20.2°, corresponding to the typical (101) reflection of semi‐crystalline PVA.^[^ ^38^ ^]^ The slight rightward shift of the peak may be influenced by the presence of CNTs.^[^ ^39^, ^40^ ^]^ Additionally, smaller peaks at 2θ = 16.5°, 19.2°, and 22.6° were observed in the BDH, indicating a higher degree of crystallinity compared to FT and FS hydrogels. This trend became more pronounced when the BDH was stretched to three times its original length. The increased crystalline domains in BDH were further corroborated by complementary XRD measurements (Figure S8, Supporting Information). Furthermore, the crystalline domains in BDH exhibited highly oriented microstructures (Figure 3f).

To further understand the alignment behavior of PVA chains during the spinning process, coarse‐grained molecular dynamics simulations were performed under both shear and elongational flow conditions. Chain orientation was quantified using the end‐to‐end vector, with the *x*‐axis corresponding to the flow direction (Figure 3e,f; Figure S9, Supporting Information). The results show that while both types of flow contribute to chain alignment, elongational flow in the coagulation bath plays a dominant role in inducing and enhancing polymer orientation. As the elongation proceeds, polymer chains become increasingly aligned. These findings are consistent with both the experimental spinning parameters and the anisotropic structures observed in SEM and WAXS, demonstrating strong consistency between simulation and experimental results.

In addition, in situ small‐angle X‐ray scattering (SAXS) was employed to further analyze the crystalline morphology of BDH during the stretching process (Figure 3g; Figure S10, Supporting Information). The spindle‐shaped SAXS patterns indicated a high degree of orientation of the nanofibers in the BDHs (Figure S10c, Supporting Information), which was driven by flow‐induced alignment. When a 300% tensile strain (BDH300%) was applied, more distinct peaks appeared in the 2D SAXS diffraction patterns (Figure S10d, Supporting Information), suggesting that the microstructure became increasingly aligned after stretching.^[^ ^41^ ^]^ Due to its higher flux and resolution, synchrotron SAXS was employed for precise characterization of nanoscale structural rearrangements (Figure 3g). The same conclusion was also observed in the WAXS scattering patterns shown in Figure 3c,d. To visually examine the microstructure of the gel, atomic force microscopy (AFM) was performed. AFM images revealed that the morphology of FT and FS hydrogels was irregular, while the BDHs exhibited a well‐oriented morphology (Figure S11, Supporting Information).

To achieve a more detailed analysis of crystalline domain structures, synchrotron SAXS was employed to obtain 2D scattering patterns with higher flux and resolution. Under uniform stretching, the SAXS results revealed 2D elliptical scattering patterns was observed, indicating a gradual increase in orientation during stretching. By integrating the 2D SAXS patterns, the SAXS curves of BDH in its initial state and at different stretching lengths were obtained (Figure 3g). As shown in Figure 3h, the peak position of the intensity curve for the BDHs shifted slightly with increasing stretching distance, suggesting stronger interference between adjacent domains. Based on Bragg's equation, the average spacing between adjacent crystalline domains was calculated from the scattering vector at the peak position (q_max_), showing a slight increase during stretching (Figure S12, Supporting Information). This trend is a typical transformation in semicrystalline polymers during the stretching process, which is associated with the mechanism of structural toughening.

### 2.4 Mechanical and Electrical Characterization of Bioinspired Directional Hydrogels

**Figure 4:**
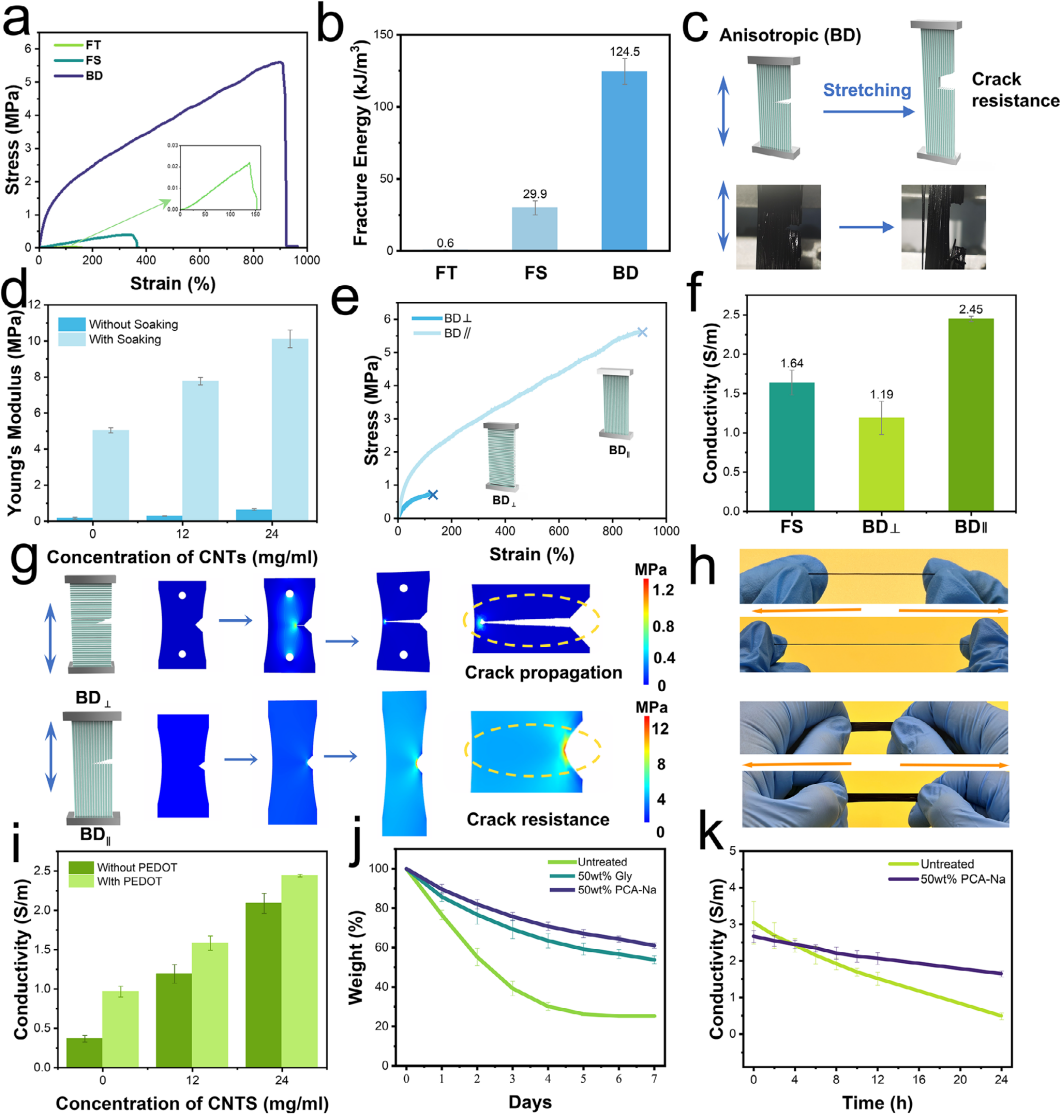
Comprehensive performance characterization of different hydrogels. a) Tensile stress‐strain curves and (b) Fracture energy of FT, FS, and BD hydrogels. c) Crack resistance of BDH. d) Young's modulus of BDH as a function of CNT content. e) Stress‐strain curves and f) Conductivity of BDH in parallel and perpendicular fiber directions. g) Crack propagation mechanisms and stress nephogram of BDH in parallel and perpendicular fiber directions. h) Optical images of single fibers and BDH under tensile stress. i) Electrical conductivity of BDH as a function of CNT content, with and without PEDOT. j) Moisture loss curve of BDH without treatment and treated with glycerol and PCA‐Na. k) Conductivity of BDH with or without PCA‐Na treatment.

To comprehensively evaluate as‐prepared BDHs, we further conducted an extensive series of measurements focusing on its mechanical, electrical, and moisture retention properties (Figure S13, Supporting Information). The mechanical performance of the PVA‐based conductive hydrogels underscored the direct correlation between their structure and functional properties. To validate the synergistic effects of flow‐induced alignment and salting out, different PVA hydrogels prepared via freezing–thawing (FT) and freezing–soaking (FS) were employed as control samples. These controls, which lacked the aligned structure or high crystallinity of BDHs, exhibited markedly lower mechanical strength (**Figure** **4**a). Notably, BDHs subjected to a 30‐min salting‐out treatment demonstrated significantly enhanced mechanical properties compared to FS hydrogels treated under identical conditions. This observation suggests that the unique fibrous architecture of BDHs facilitates rapid ion diffusion within the polymer network, thereby promoting swift aggregation and crystallization. Moreover, BDHs exhibited superior crack resistance compared to FT and FS hydrogels (Figure 4b). While FT and FS hydrogels were prone to microcrack formation and propagation under stress, BDHs effectively suppressed crack growth due to their unique fibrous network and enhanced crystallinity. The aligned nanofibrillar structures in BDHs facilitated efficient stress dissipation, preventing localized stress concentration at crack tips (Figure 4c; Figure S14, Supporting Information).

Tensile tests were also performed on hydrogels incorporating varying concentrations of PC‐ink, both with and without subsequent salt soaking treatment (Figure 4d). According to literature reports, once the CNT content exceeds a certain threshold, its effect on mechanical properties shifts from beneficial to detrimental.^[^ ^35^ ^]^ Moreover, an excessively high CNT concentration can result in polymer chain entanglement, potentially clogging the syringe and disrupting the wet spinning process, which in turn yields non‐uniform fibrous structures. Consequently, an optimal concentration range was identified for the PDA‐CNT ink (0–24 mg mL^−1^). Within this range, the hydrogels exhibited excellent mechanical properties that improved progressively with increasing PDA‐CNT concentration. Additionally, the Young's modulus of the hydrogels increased significantly after a 30‐min immersion in the PCA‐Na solution—a change attributed to enhanced crystallization among the nanofiber chains within the hydrogel network.^[^ ^42^, ^43^ ^]^

Beyond its role in enhancing mechanical integrity, PCA‐Na proved effective in mitigating dehydration of the BDHs under ambient conditions—a critical factor for preserving both mechanical performance and electrical conductivity. As illustrated in Figure 4e, untreated hydrogels experienced significant dehydration, leading to weight loss and a decline in both mechanical and electrical properties. In contrast, the incorporation of PCA‐Na substantially reduced dehydration‐related weight loss, outperforming hydrogels soaked in glycerol at equivalent concentrations. The exceptional water retention capability can be attributed to two synergistic mechanisms: 1) the formation of robust hydrogen bonds between PCA‐Na and water molecules, which creates a water‐locking effect that significantly inhibits evaporation, and 2) the progressive replacement of free water molecules by PCA‐Na within the polymeric matrix. This substitutional effect is particularly pronounced at higher PCA‐Na concentrations, where the compound's intrinsically lower vapor pressure establishes an effective barrier against water loss, thereby substantially reducing the evaporation rate.

After determining the precursor composition and fabrication method of the BDHs, we further investigated how the anisotropic structure influences their mechanical and electrical performance along different directions. As shown in Figure 4e, the stress–strain curves exhibit a significant difference between the directions parallel and perpendicular to the fiber alignment. This difference originates from the structural anisotropy: the mechanical response in the parallel direction reflects the collective behavior of the aligned gel matrix, while the response in the perpendicular direction is dominated by the limited resistance offered by the interstitial regions between fibers. This directional dependence is reminiscent of the structural mechanics of oriented biological tissues such as muscles or tendons, highlighting the bioinspired design principle.

The electrical conductivity of the BDHs along both directions was also evaluated (Figure 4f). In contrast to the isotropic FS hydrogels, the BDHs displayed clear anisotropy in conductivity. This behavior is likely attributed to the directional arrangement of conductive fillers, which could form more efficient percolation pathways along the fiber alignment. Furthermore, macroscopic structural factors, such as the presence or absence of voids in each direction, may also contribute to the observed differences in conductivity.

To gain further insight, a finite element simulation was performed to analyze crack propagation behavior in both directions (Figure 4g). Precut cracks were introduced perpendicular to the tensile axis before stretching. The ordered fiber architecture endowed the BDHs with damage tolerance, enabling enhanced crack resistance in the parallel direction, while rapid crack propagation was observed in the perpendicular direction. These findings are consistent with the experimental stress–strain results (Figure 4h). Together, both experimental and theoretical analyses underscore the functional role of the fiber‐aligned structure in resisting crack propagation and producing direction‐dependent electrical responses, echoing the features of their biological counterparts.

While the BDHs exhibit anisotropic conductivity across different orientations, the electrical conductivity of the BDHs is predominantly governed by the CNT concentration in the ink. As shown in Figure 4i, hydrogels prepared with 24 mg mL^−1^ CNT ink exhibited electrical conductivity ≈5 times higher than those prepared with pure water. This improvement is attributed to the formation of well‐connected conductive pathways facilitated by the CNT network. However, beyond an optimal concentration, excessive CNTs can lead to agglomeration and diminished dispersion, thus limiting further improvements in conductivity. To further enhance conductivity, PEDOT:PSS was incorporated to create more interconnections between CNTs, effectively lowering the overall electrical resistance (Figure S15, Supporting Information). Additionally, the saline solutions employed during hydrogel preparation introduced salt ions into the network, which not only promoted crystallization and mechanical reinforcement but also contributed additional ionic pathways, collectively enhancing the electrical conductivity.

As mentioned above, moisture retention plays a crucial role in the performance of conductive hydrogels, particularly for systems involving ionic conductivity. In our material design, the PCA‐Na soaking step not only enhances mechanical strength and electrical conductivity but also fundamentally serves to improve the moisture retention of BDHs. As shown in Figure 4j, BDHs treated with PCA‐Na demonstrate superior water retention over extended periods compared to untreated samples at the same concentration. To further investigate this effect, the electrical conductivity of BDHs was monitored over time. Interestingly, while the untreated BDHs initially exhibited higher conductivity due to their higher water content, a rapid decrease in conductivity was observed, and by the 4‐hour mark, the conductivity had already dropped below that of the BDHs treated with PCA‐Na (Figure 4k). This behavior highlights the critical role of moisture retention in ensuring the long‐term stability of the electrical performance in conductive hydrogel systems.

The incorporation of PCA‐Na also contributes to the long‐term mechanical stability of BDH. The BDH treated with PCA‐Na maintains consistent mechanical performance under repeated stretching, showing minimal energy dissipation and negligible degradation over 100 cycles at 10% strain (Figure S16a, Supporting Information). While a slight decrease in stress amplitude is observed, the slope of the stress–strain curve remains nearly unchanged, indicating preserved stiffness and structural integrity. In contrast, the control hydrogel without moisturizing treatment exhibits a significantly lower initial Young's modulus and a gradual reduction in stress response during cycling (Figure S16b, Supporting Information). More notably, by the 100th cycle, the slope of its stress–strain curve increases, suggesting drying‐induced stiffening and compromised mechanical resilience.

In addition, to assess the hydrogel's electrical response to mechanical stress, tensile and resistivity tests were further conducted (Figure S17, Supporting Information). When the sample was stretched by 80%, its resistance increased to ≈160% of the initial value. During cyclic loading and unloading, the sensor exhibited a remarkably short response time (490 ms) and recovery time (360 ms). Moreover, the progressive changes in relative resistance with increasing strain underscore the BDH's potential as a stress‐sensitive resistive sensor capable of providing real‐time, targeted feedback.

### 2.5 Enhancement of Jump Performance in Sports Training

The coordinated movement capabilities of the knee and surrounding muscles are particularly crucial for individuals engaged in basketball training, as they directly influence explosive performance and play a central role in injury prevention. The knee, being a critical weight‐bearing and stabilizing joint, is subjected to high levels of stress during basketball‐related activities such as jumping, landing, and rapid directional changes. These motions not only test the lower limb strength but also pose risks for overuse and acute injuries. Vertical jumping, a fundamental component of basketball training, is widely used to assess athletic performance and neuromuscular coordination. In this study, motion signals around the knee joint were collected from a subject engaged in basketball exercises, who wore BDHs positioned on the front of the knee while performing a series of actions, such as jump training, simulating real basketball training scenarios.

**Figure 5:**
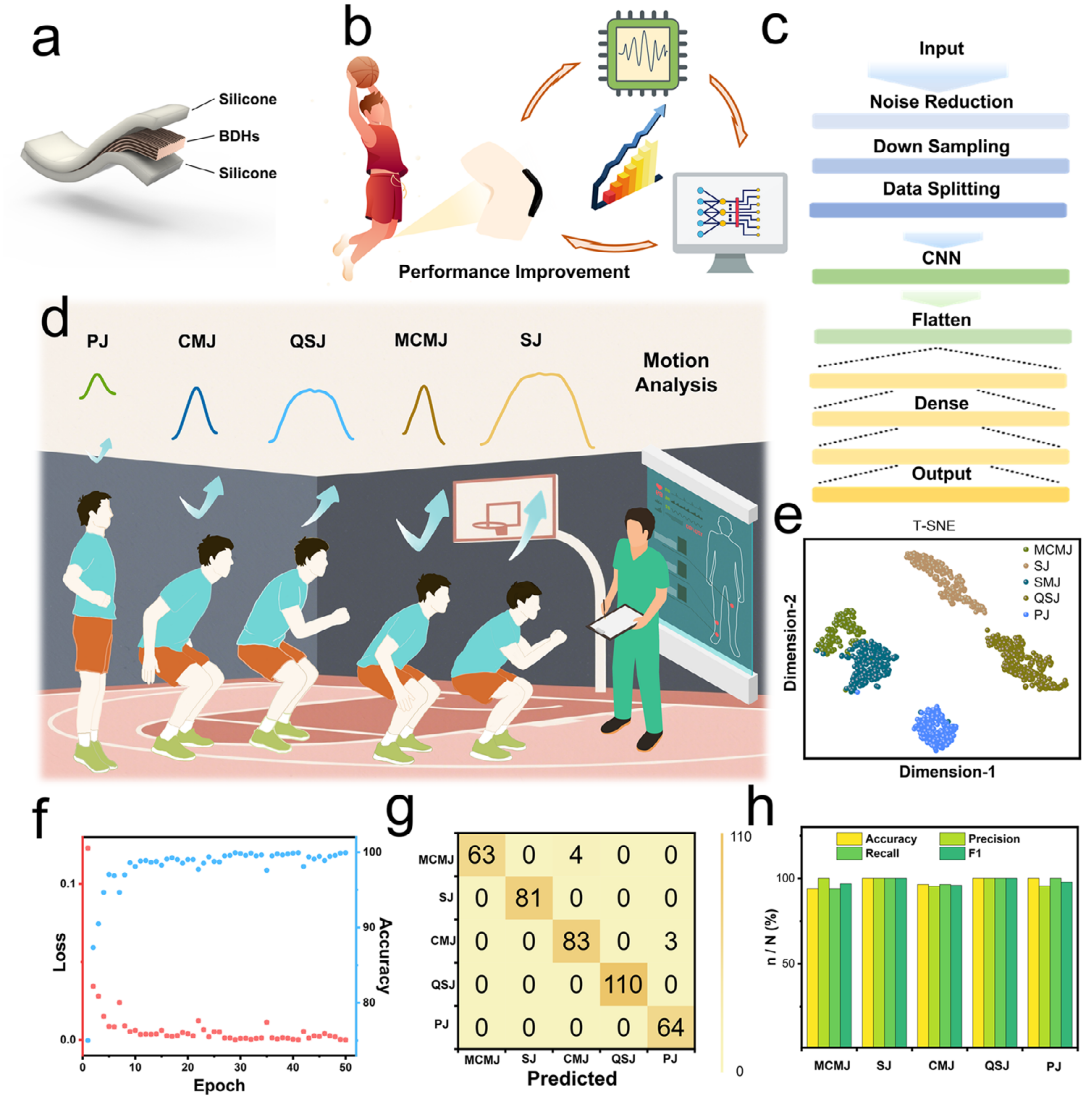
Performance evaluation of BDH‐based sensor application in jumping motion intention recognition via deep learning. a) Schematic illustration of BDH sensor encapsulation. b) Demonstration of BDH application in enhanced motion training. c) Schematic of the deep learning model used for motion analysis. d) Representative signals recorded by BDHs during pre‐jump preparation. e) t‐SNE visualization of model‐generated feature outputs. f) Loss and accuracy curves over 50 training iterations. g) Confusion matrix showing the classification performance of the model. h) Performance metrics (Accuracy, Precision, Recall, and F1‐score) for model evaluation.

To ensure reliable performance under practical conditions where sensors are subjected to significant mechanical strain during high‐intensity movements, our as‐prepared BDHs demonstrate superior strain tolerance compared to non‐aligned hydrogels (Figure S18, Supporting Information). In addition, to enhance the wearability of BDHs and improve their stability and friction on the skin surface during sweating, the as‐prepared BDHs were further encapsulated with a layer of silicone (**Figure** **5**a; Figure S19, Supporting Information). The encapsulated device was then connected to the electronic circuit and secured to the front of the subject's knee using a sports knee brace. During basketball‐related training exercises, the collected signals were processed and used to accurately classify completed movements, helping to evaluate and improve training precision and effectiveness (Figure 5b).

In principle, accurate motion recognition depends on the quality of signal feature extraction, segmentation, and classification. The corresponding algorithms include signal processing techniques, position segmentation algorithms, and convolutional neural network (CNN)‐based classification models. The processing and prediction pipeline is systematically outlined in Figure 5c. First, the raw signals are filtered and denoised to eliminate outliers and enhance signal clarity. The data is then downsampled from 1000 to 50 Hz to increase computational efficiency while maintaining key signal characteristics. A position segmentation algorithm was then used to detect knee flexion patterns associated with jumping motions, selecting a 2.5‐s time window before and after each voltage peak for further analysis.

Due to the symmetric nature of stretching, multiple jump pattern detection in the same subject requires only the strain response signal of a single lower limb for full classification identification, as shown in Figure 5d. According to conventional training actions in targeting different aspects (e.g., reactive strength and explosive power), jumping movements in this work are categorized into five types: 1) Pogo jump (PJ), 2) Countermovement jump (CMJ), 3) Quanter‐squat jump (QSJ), 4) Maximal effort countermovement jump (MCMJ), and 5) Squat jump (SJ) (Figure 5d; Figure S20, Supporting Information). T‐distributed stochastic neighbor embedding (t‐SNE) was used to reduce the dimensionality of feature parameters, allowing the knee motion signals to be visualized as a point in a 2D plane.^[^ ^44^ ^]^ It is clear that the different gesture datasets, represented by clusters of different colors, show significant differences (Figure 5e; Figure S20, Supporting Information).

Although the electrical signals generated by jumping movements appear visually different, they cannot be accurately identified directly. Hierarchical feature extraction through CNNs was employed for classification. In this work, 64% of the entire dataset was used for training, with 16% of that was reserved for validation. The remaining 20% of the dataset was used as the test set to assess the model's accuracy. As training progressed, the accuracy increased, indicating that the model was effectively classifying training samples (Figure 5f). Meanwhile, the loss function gradually decreased and stabilized, confirming that the model reduced prediction errors and avoided overfitting during optimization. After 50 epochs, our proposed model achieves 98.2% accuracy in classifying five different jumping patterns (Figure S21, Supporting Information). Figure 5g presents the confusion matrix of the gesture recognition results, where each column represents the predicted jumping patterns and each row corresponds to the actual jumping patterns. Meanwhile, the consistency of high accuracy, precision, recall, and F1 score confirms the comprehensive capability of the model in both classification and generalization (Figure 5h). These results demonstrate that integrating biomechanical parameters with deep learning enabled precise classification of motion signals. More importantly, this approach provides a reliable method for recognizing and assessing jump‐related actions, such as basketball training movements, thereby enabling objective evaluation of athletic ability and performance.

## 3 Conclusion

In summary, drawing inspiration from the structural organization and moisturizing functions found in biological organisms, we have developed conductive, moisture‐retentive BDHs with exceptional performance for detecting various jumping patterns. These BDHs with well‐ordered hierarchical structures were fabricated through flow‐induced alignment of PVA chains, PDA‐CNTs, and PEDOT:PSS, and followed by immersion in PCA‐Na. Due to the synergistic effects of hierarchical fiber alignment and NMFs, the resulting BDHs exhibit enhanced mechanical properties, robust electrical conductivity, and excellent moisture retention, making them well‐suited for sensors that must withstand high stress. When integrated with deep learning algorithms, these BDHs demonstrate the capability for accurate motion pattern recognition and classification, supporting their applications in athletic training assistance and rehabilitation monitoring. This approach shows strong potential in fields such as sports science, rehabilitation, and human‐computer interaction, especially in supporting structured movement analysis and performance optimization. Previous work on wearable sensors with carefully engineered device architectures has provided valuable insights into achieving high‐performance signal detection and stability in dynamic environments,^[^ ^45^ ^]^ which may guide the continued development of BDH‐based systems for broader practical applications by optimizing their device structures and functionalities with specific sensing requirements.

## 4 Experimental Section

### Materials

Polyvinyl alcohol (PVA, M.W. 146000–186000, Sigma–Aldrich), multi‐walled carbon nanotubes (Chengdu Organic Chemicals Co.), Dopamine hydrochloride (98%, Macklin), PEDOT:PSS (Clevios P VP Al 4083, from Heraeus, J&K Scientific Co.), ammonium sulfate (Xilong Scientific Co.), sodium pyrrolidone carboxylate acid (PCA‐Na, AR, 50%, Macklin) and Tris‐HCl buffer solution (10 mM, pH = 8.5, MeilunBio) were purchased and used without further purification.

### Preparation of PDA‐CNT Electroconductive Ink

MWCNTs (1200 mg) were dissolved in the 100 mL of Tris‐HCl buffer solution. The mixture was sonicated for 30 min under 750 W power using a probe sonicator (VCX750, SONICS & MATERIALS, INC.). Subsequently, dopamine hydrochloride powder (1800 mg) was added to the above mixture, and the reaction lasted for 24 h under continuous mechanical stirring. Finally, after centrifugation and washing, the precipitate was diluted to 50 mL with deionized water.

### Preparation of Conductive Fibrous Hydrogels

1 g of PVA and 1 mL of PEDOT:PSS were added to 8 mL of the PDA‐CNT electroconductive ink with different concentrations. The mixtures were heated to 90 °C and vigorously stirred for 2 h. After standing for 1 h to vent, the homogeneous solutions were obtained. This precursor solution (10 mL) was loaded into a syringe and injected into a 40 wt.% ammonium sulfate solution (injection speed: 0.6 mL min^−1^) by a syringe pump, passing through a coagulation bath less than 30 cm to obtain hydrogel fibers. The spun hydrogel fibers were collected and assembled by a servo‐motor‐driven reeling bobbin (diameter: 40 mm, rotation speed: 25 rpm). The bobbins with hydrogel fiber were immersed in PCA‐Na solution for 30 min. Finally, the resulting fibrous hydrogel was cut and removed to obtain bioinspired direction hydrogels. As a comparison, Isotropic hydrogels were prepared with the same precursor solution, which was poured into Teflon molds and frozen at −20 °C. After 8 h, the frozen samples were thawed at room temperature for 3 h to obtain FT hydrogels and immersed in salt solution to obtain FS hydrogels.

### Characterization of PDA‐CNT

The adsorption of PDA on the surface of CNTs was investigated by transmission electron microscopy (JEM 2100F, JEOL). The samples were prepared by dropping the prepared aqueous dispersion onto the amorphous carbon‐coated copper grids and dried. XPS spectra were obtained using K‐Alpha from Thermo Fisher Scientific. The neutral C1s peak (C─C(H), set at 284.6 eV) was used as a reference for charge correction. FTIR spectroscopy was conducted with 64 scans at a resolution of 0.4 cm^−1^, and the wavenumber range for 4000–1000 cm^−1^ for each measurement with a Nicolet IN10 spectrometer (Thermo Fisher Scientific, USA). The unmodified CNTs underwent identical sonication and washing procedures as the control group.

### SEM Characterization

To investigate the structure and surface morphology of the BDHs, samples were frozen with liquid nitrogen and then freeze‐dried. Then, the lyophilized hydrogels were sputtered with gold and observed using SEM (ΣIGMA‐HD, ZEISS) at an acceleration voltage of 8 kV.

### AFM Imaging

FT hydrogel, FS hydrogel, and BDH were air‐dried in an incubator at 37 °C and subsequently characterized by AFM in tapping mode for surface morphology. In this mode, the probe lightly tapped the surface, and the height of the sample surface was recorded.

### X‐Ray Scattering Characterization

The wide‐angle X‐ray scattering (WAXS) measurements were performed at Xeuss 2.0 SAXS System (Xenocs, France). The in situ small‐angle X‐ray scattering (SAXS) measurements were conducted both in the laboratory and at the synchrotron facility to analyze the structural evolution of BDH under stretching. The laboratory SAXS measurements were carried out at Xeuss 2.0 SAXS System (Xenocs, France). The in situ SAXS measurements were carried out at BL16B1 beamline (Shanghai Synchrotron Radiation Facility). The light source had a wavelength of 0.124 nm and the sample‐to‐detector distance was 2188 mm. Sas‐View software was adopted to fit the SAXS results to estimate the average distance between adjacent crystalline domains (D_ac_) and the average size of crystalline domains (D_c_).

### Molecular Dynamics Simulation of the Spinning Process

The long PVA chain was coarse‐grained, turned into a chain made up of 100 particles that represent the short chain containing 33 monomers. Water molecular are also coarse‐grained as small water droplets possessing the same radius of σ_0_ = 3nm^[^ ^28^ ^]^ with the polymer coarse‐grain beads. The interaction between different particles was described as below:

For particles on the chain, the bond potential is set with FENE.^[^ ^27^ ^]^

(1) $$E=-0.5KR_{0}^{2}\ln\left[1-{\left(\frac{r}{R_{0}}\right)}^{2}\right]+4\varepsilon \left[{\left(\frac{\sigma }{r}\right)}^{12}-{\left(\frac{\sigma }{r}\right)}^{6}\right]+\varepsilon$$

For non‐bond interaction, the Lennard‐Jones potential was used with a cut off distance.

(2) $$E=4\varepsilon \left[{\left(\frac{\sigma }{r}\right)}^{12}-{\left(\frac{\sigma }{r}\right)}^{6}\right],r<r_{c}$$

For LJ potential parameter, ε was set between the same kind of particles with 0.5, 0.6 for LJ potential between different types of particles, for more parameter configurations see the essay.^[^ ^26^ ^]^

The vector starting at one end of and ending at the other end of the same polymer chain was used to show the effect of elongation and shear on the chain, and calculate the average value of the vector and its length.

(3) $$\vec{R_{i}}=\vec{r_{hi}}\;-\vec{r_{ti}}$$

(4) $$<\vec{R}\geq \frac{\sum_{i=1}^{N}\vec{R_{i}}}{N}$$

(5) $$<\left|R\right|\geq \frac{\sum_{i=1}^{N}\left|R_{i}\right|}{N}$$

LAMMPS was used for the simulation. Ovito was used for the visualization. The post‐process of simulation data is carried out through Python and relevant open‐source libraries.

### Mechanical Tests

The mechanical properties of the hydrogel were tested using a universal tensile machine (JHY‐5000) during tensile tests and continuous loading. The stretching speed was set as 50 mm/min in the tensile test. The strain was obtained as the ratio of the change in length to initial length, and stress was obtained by dividing the force by the cross‐sectional area of the sample. Young's modulus was estimated from the slope of the initial linear region of the stress‐strain curve. Pure shear tests were conducted to calculate the fracture energy of the hydrogel. Unnotched and notched samples with identical initial dimensions were stretched, with the notch size approximately half the width of the sample. The fracture energy is calculated with the formula Γ = U(Lc) / A, where Lc denotes the critical extension at which the notch propagates into a running crack, U(Lc) represents the work required to extend the unnotched sample to Lc, and A is the cross‐sectional area of the hydrogel.

### Conductivity Measurements

The samples were processed into homogeneous rectangles, and the resistance was measured as if the samples were perfect rectangular solids. The measurements were performed at room temperature and humidity. The conductivities of hydrogels (σ) were calculated as: σ = 𝐿 /𝑅×S, where L was the distance between the electrodes, R was the output resistance, and S was the cross‐sectional area of the sample.

To minimize the mechanical disturbance from the measurement setup during transverse conductivity testing, an insulating tape was placed beneath the sample as a buffer layer.

### Measurement of Crystallinity

The crystallinity of the hydrogels was measured in their dry state by a differential scanning calorimeter (DSC, NETZSCH). To minimize the formation of further crystalline domains during the air‐drying process, excess chemical cross‐links were used before. Initially, the hydrogels were soaked in a 100 mL solution containing 10 mL of glutaraldehyde (50% in H_2_O) and 1 mL of hydrochloric acid (36.5–38 wt.%) for 6 h. Afterward, they were soaked in deionized water for 24 h to remove excess chemicals. The treated samples were then dried in an incubator at 37 °C for 2 h.

Thereafter, the mass of residual water m_residual_, the mass of crystalline domains m _crystalline_, and the total mass of the dry samples (with residual water) m were measured. In a typical DSC measurement, the total mass of the dry sample was weighed first. Then the sample was placed into a Tzero pan and heated from 50 to 250 °C at a rate of 20 °C/min under a nitrogen atmosphere. The DSC curves revealed a broad peak between 60 and 180 °C, corresponding to the residual water in the sample. Using the method described in previous studies (Lin et al. 2019), the mass of the residual water m_residual_ was calculated as m_residual_ = m_initial_ · H_residual_ / H^0^_water_, where m_initial_ is the mass of the sample before DSC testing, and H^0^_water_ = 2260 J/g is the latent heat of water evaporation. Additionally, a narrow peak was observed between 200 and 250 °C, corresponding to the melting of the PVA crystalline domains. The mass of the crystalline domains m_crystalline_ was determined by the equation m_crystalline_ = m_initial_ · H_crystalline_ / H°_crystalline_, where H°_crystalline_ = 138.6 J/g is the enthalpy of fusion of fully crystalline PVA measured at the equilibrium melting point (Peppas and Merrill 1976). Based on these values, the crystallinity in the dry state was calculated as X_dry_ = m_crystalline_ / (m_initial_ – m_residual_). To measure the crystallinity in the swollen state, the swollen hydrogels were weighted m_swollen_ and weighted m after air‐drying in an incubator at 37 °C for 8 h.

The water content in swollen state W can be calculated as (m_swollen_ + m – m_residual_)/ m_swollen_, and the crystallinity in the swollen state was calculated as X_swollen_ = X_dry_ · (1 – W), where W represents the water content of the sample.

### Water Retention Rate Testing

With the other preparation steps being the same, the hydrogel fiber was directly cut and removed from the reel as the untreated hydrogels, while that was soaked with bobbins in glycerol for 30 min before getting cut and removed to obtain the glycerol hydrogels. The samples were placed in an environment where the temperature was 25 °C and the humidity was 40%.

## Author Contributions

H.W. and S.W. contributed equally to this work. H.W. contributed to writing – original draft and review & editing, conceptualization, methodology, visualization, and investigation. S.W. was involved in conceptualization, methodology, visualization, and investigation. Y.J. contributed to methodology, software, and validation. Z.H. was responsible for data curation and investigation. D.L. and Q.L. contributed to visualization and investigation. T.F. was involved in methodology and software. H.Z. contributed to data curation and visualization. D.Y. participated in visualization and investigation. J.Y. contributed to writing, review & editing, supervision, and investigation. Y.H. was responsible for project administration, resources, supervision, and writing – review & editing. Y.L. contributed to writing – review & editing, project administration, supervision, conceptualization, visualization, and funding acquisition.

## Conflict of Interest

The authors declare no conflict of interest.

## Supporting information



Supporting Information

## Data Availability

The data that support the findings of this study are available from the corresponding author upon reasonable request.
