# Supplementary material for: Bioinspired Directional Hydrogel‐Based High‐Performance Flexible Sensor for Multiple Jumping Pattern Detection in Athletic Training
Source: Adv Sci (Weinh). 2025 Oct 20;13(1):e15261. doi: 10.1002/advs.202515261 (PMC12767052; doi:10.1002/advs.202515261)
Supplement: Supplementary file 1 — Supporting Information [file ADVS-13-e15261-s001.docx]

**Bioinspired Directional Hydrogel-based High-performance Flexible Sensor for Multiple Jumping Pattern Detection in Athletic Training**

*Hanqi Wang,^1, 2, #^, Sen Wang^2 #^,* *Yirong Jiang^2^, Zhehao Han^3^, Da Lin^2^, Qinglu Luo^4, 5^, Tianqi Fu^2^, Hanyi Zhang^6^, Deshuai Yu^2^, Jia Yi^1, 2^**^*^, Yan Hu^3^*, Youhui Lin**^1,2^**

^1^National Institute for Data Science in Health and Medicine, Xiamen University, Xiamen, 361102, P. R. China.

^2^Department of Physics, Research Institute for Biomimetics and Soft Matter, Fujian Provincial Key Laboratory for Soft Functional Materials Research, Xiamen University, Xiamen 361005, P. R. China

^3^The Department of gynecology, The First Affiliated Hospital of Wenzhou Medical University, Wenzhou, Zhejiang, 325000, P. R. China.

^4^Department of Rehabilitation, the Tenth Affiliated Hospital of Southern Medical University, Dongguan People's Hospital, Dongguan, 523000, P. R. China.

^5^Dongguan Experimental Centre for Sports Rehabilitation Research

Dongguan, 523000, P. R. China.

^6^Xiamen University Affiliated Keji High School, Xiamen 361102, P. R. China

*^#^*The authors contributed equally to this work

*Correspondence: linyouhui@xmu.edu.cn (Y.H. Lin); Drhuyan@wmu.edu.cn (Y. Hu), yijia@stu.xmu.edu.cn (J. Yi)


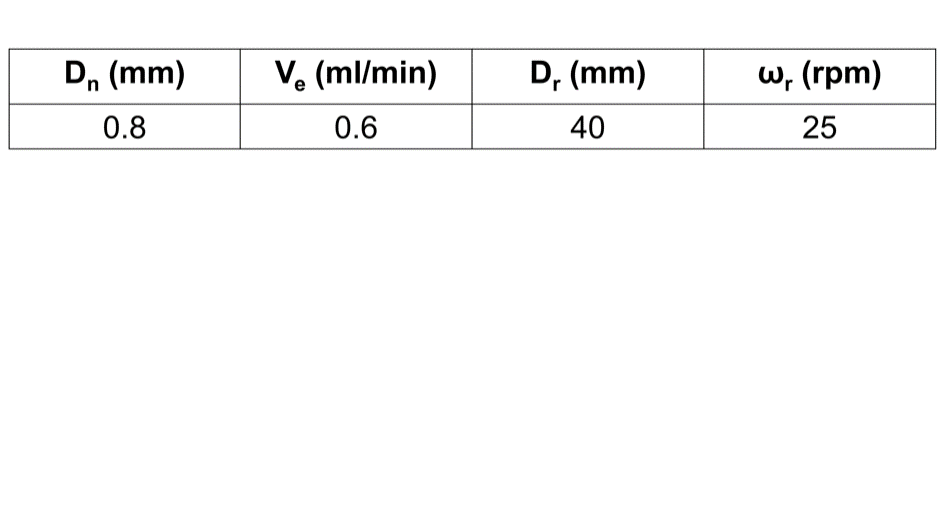


**Table S1.** Key fabrication parameters for BDHs, including needle diameter (D_n_), extrusion rate (V_e_), spool diameter (D_r_), and rotational speed of the spool (𝛚_r_).


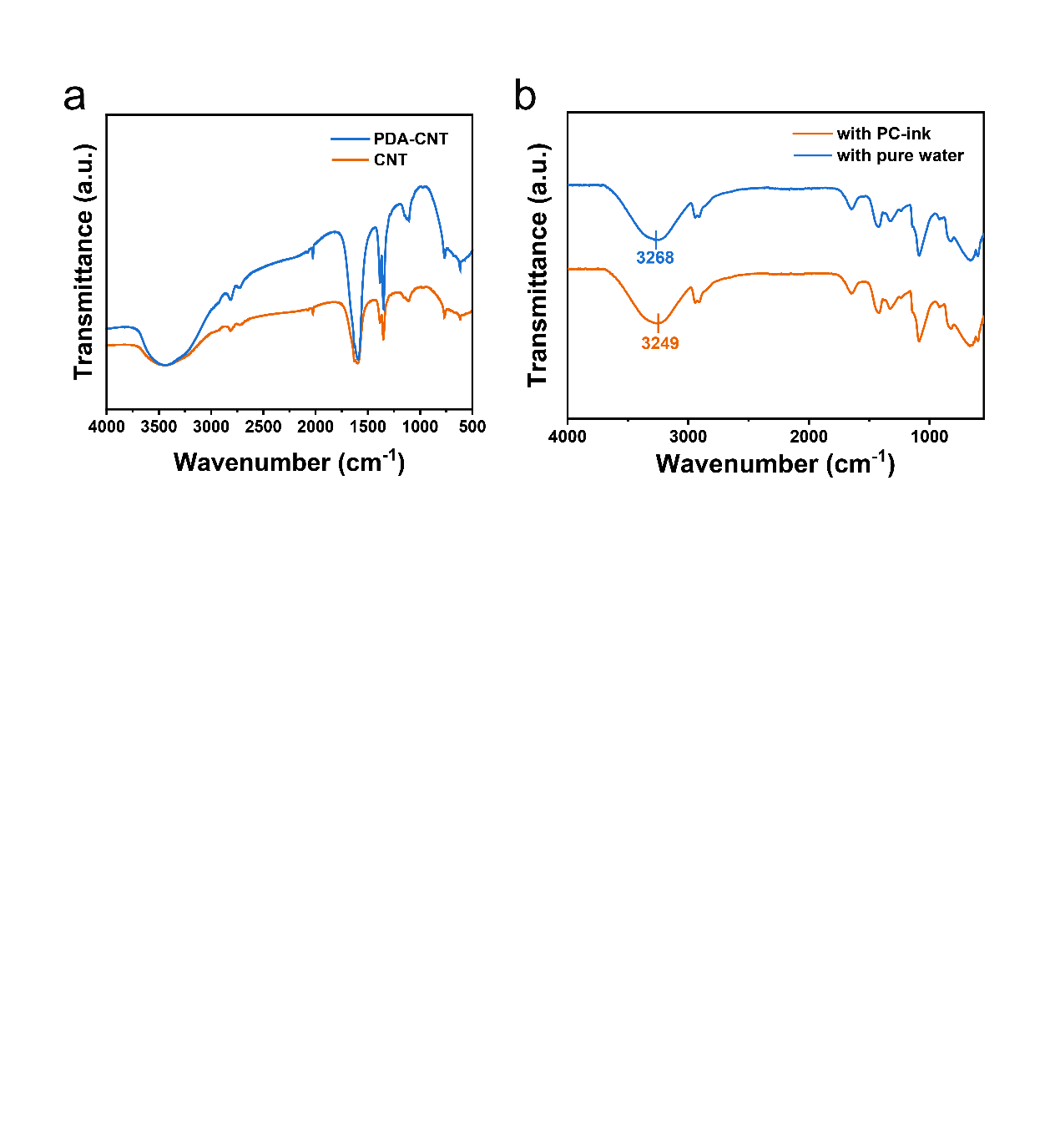


**Figure S1.** (a) FTIR spectra of CNT and PDA-CNT. (b) FTIR spectra of BDHs manufactured with PC-ink and pure water.


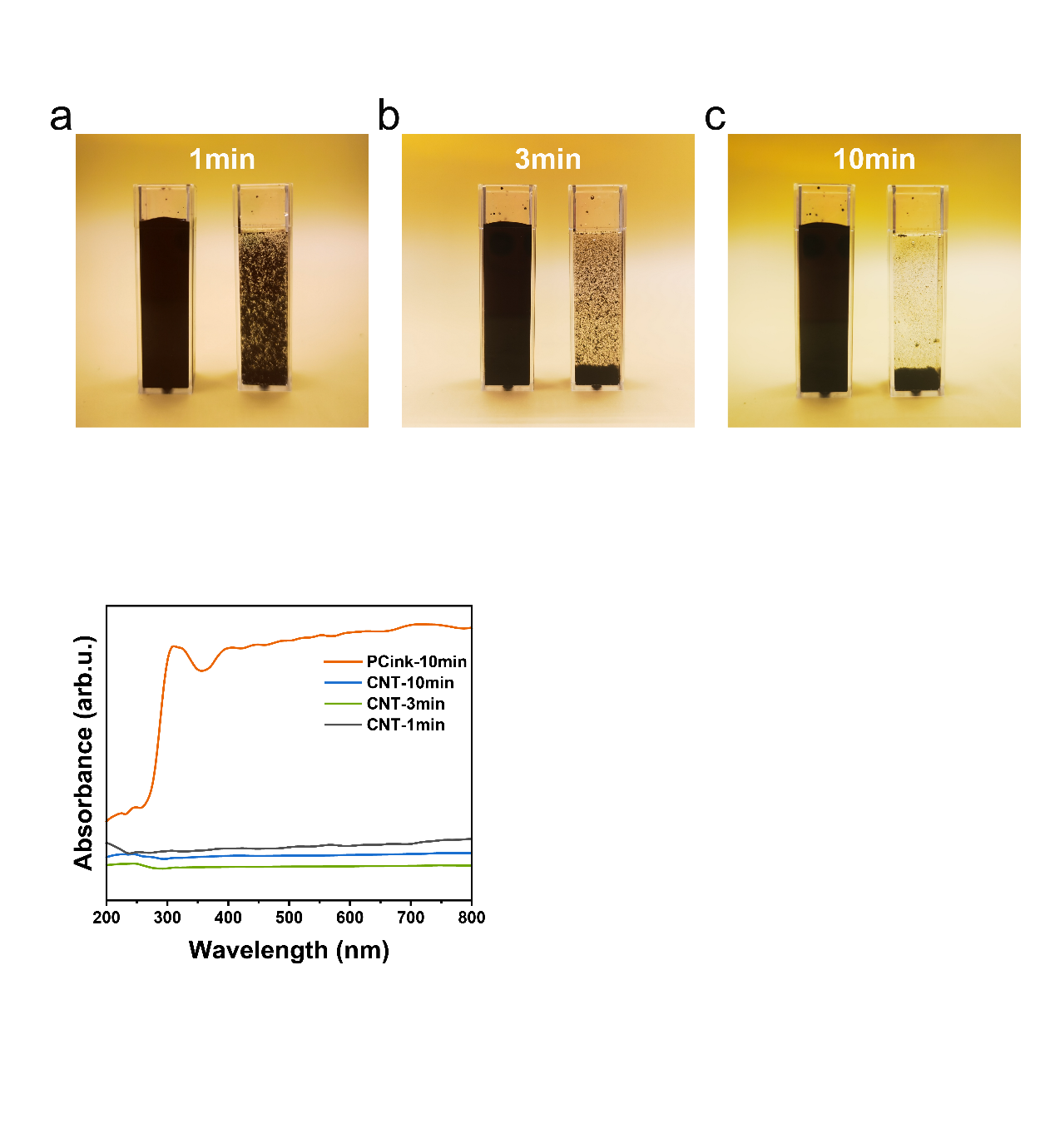


**Figure S2.** Typical photograghs of  aqueous dispersions of PDA-CNTs and CNTs after ultrasonic dispersion of 1, 2, and 10 mintutes.


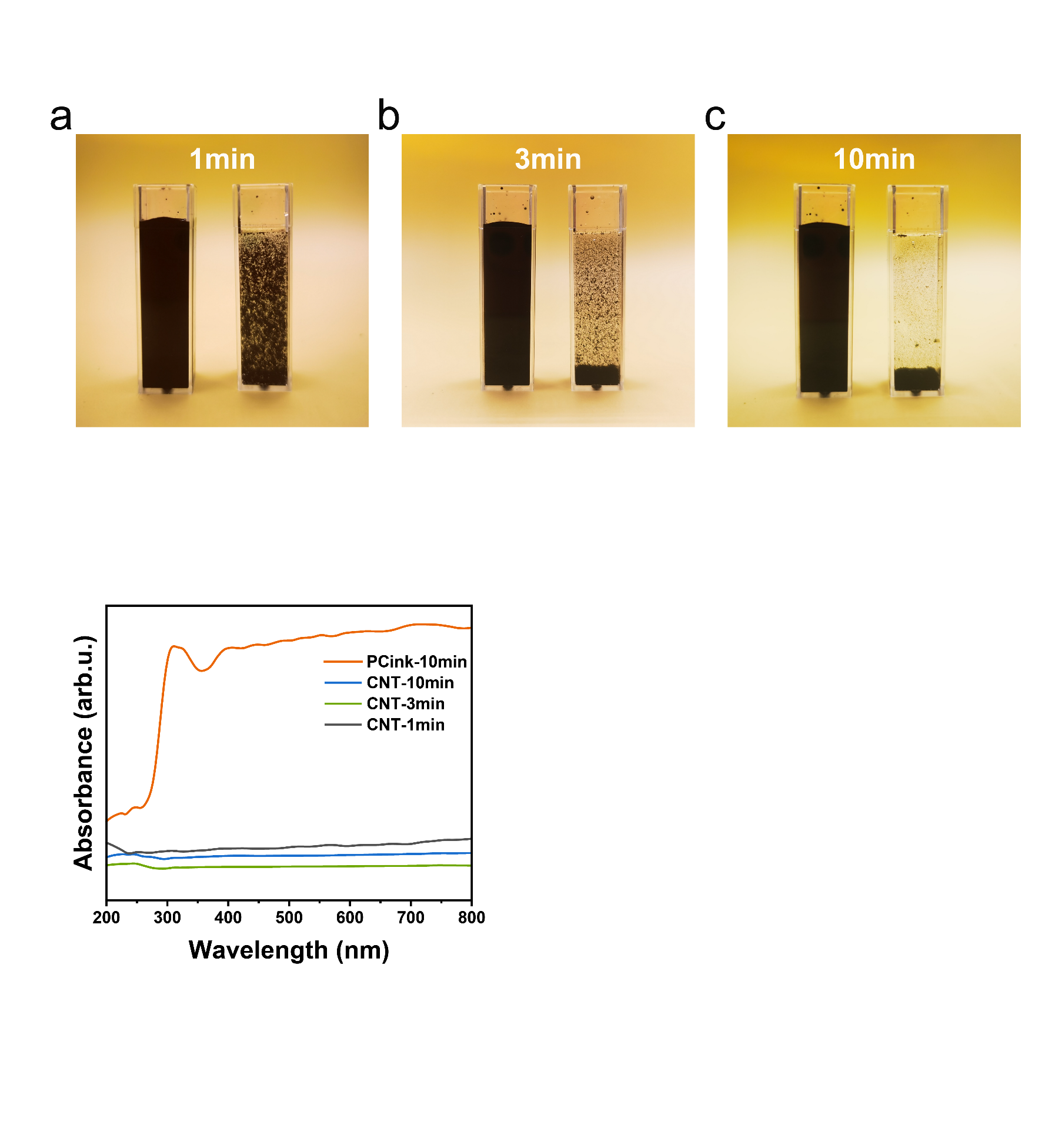


**Figure S3.** UV–vis spectra of aqueous dispersions of PDA-CNT and CNT.

**
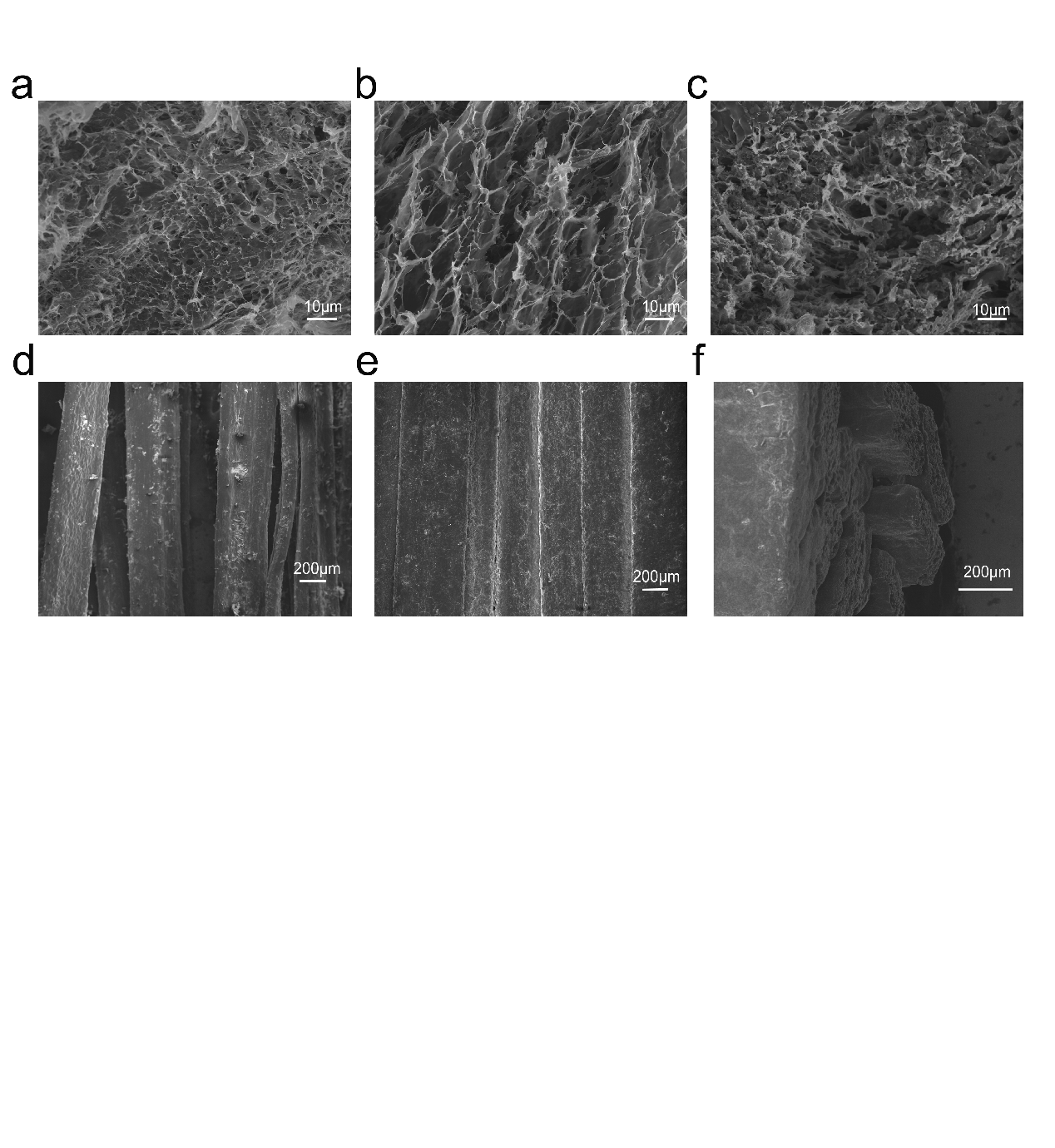
**

**Figure S4.** SEM images of (a) FT hydrogel, (b) FS hydrogel soaking with (NH_4_)_2_SO_4_ solution, (c) FS hydrogel soaking with PCA-Na solution. (d) Dispersion state of the as-extended fibers of BDHs, (e) Surface morphology of BDHs, (f) Morphology of the naturally formed end of BDHs.


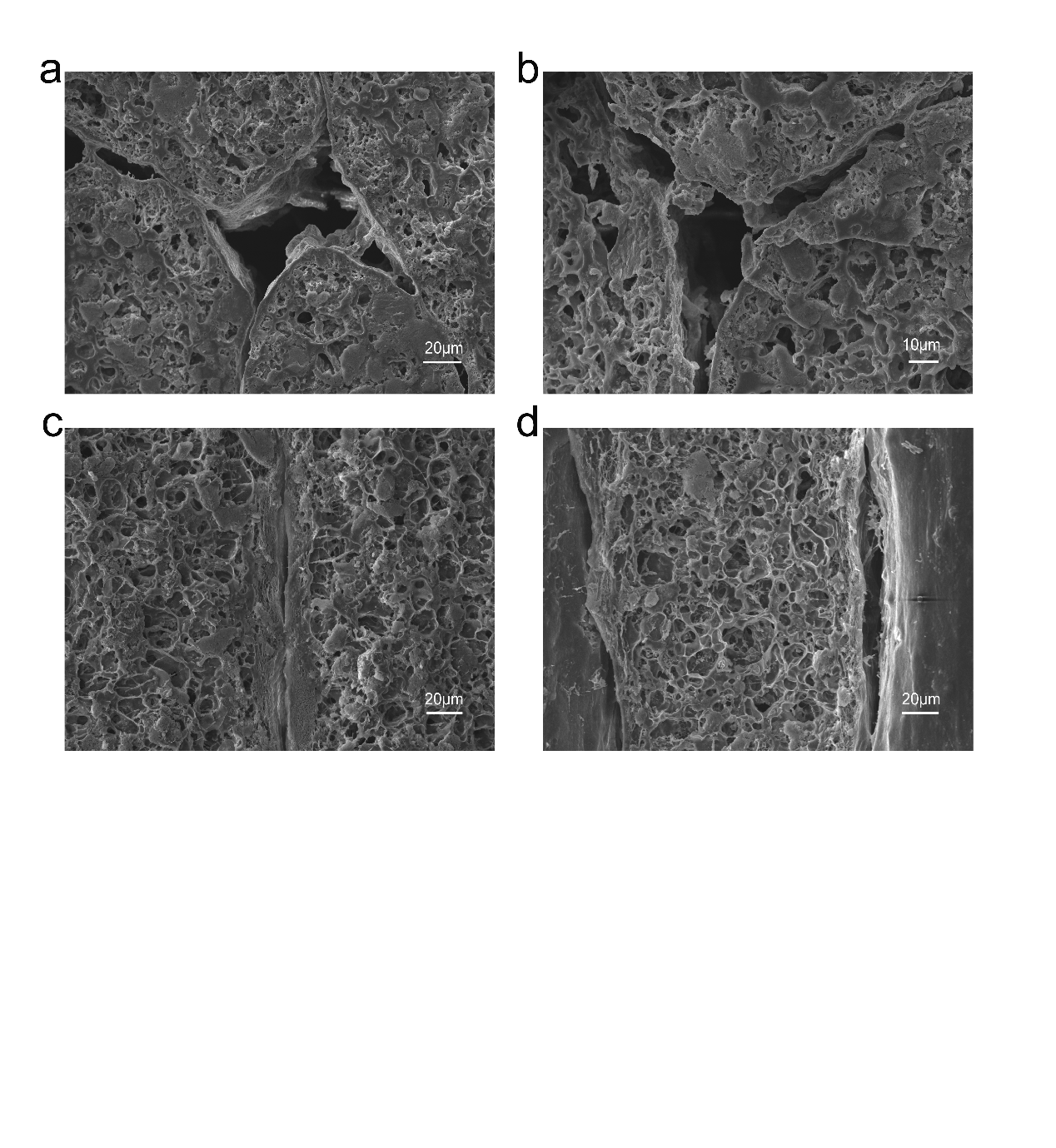


**Figure S5.** SEM images of cross-sectional BDHs. (a, b) section parallel to the fiber orientation, (c, d) section perpendicular to the fiber orientation.


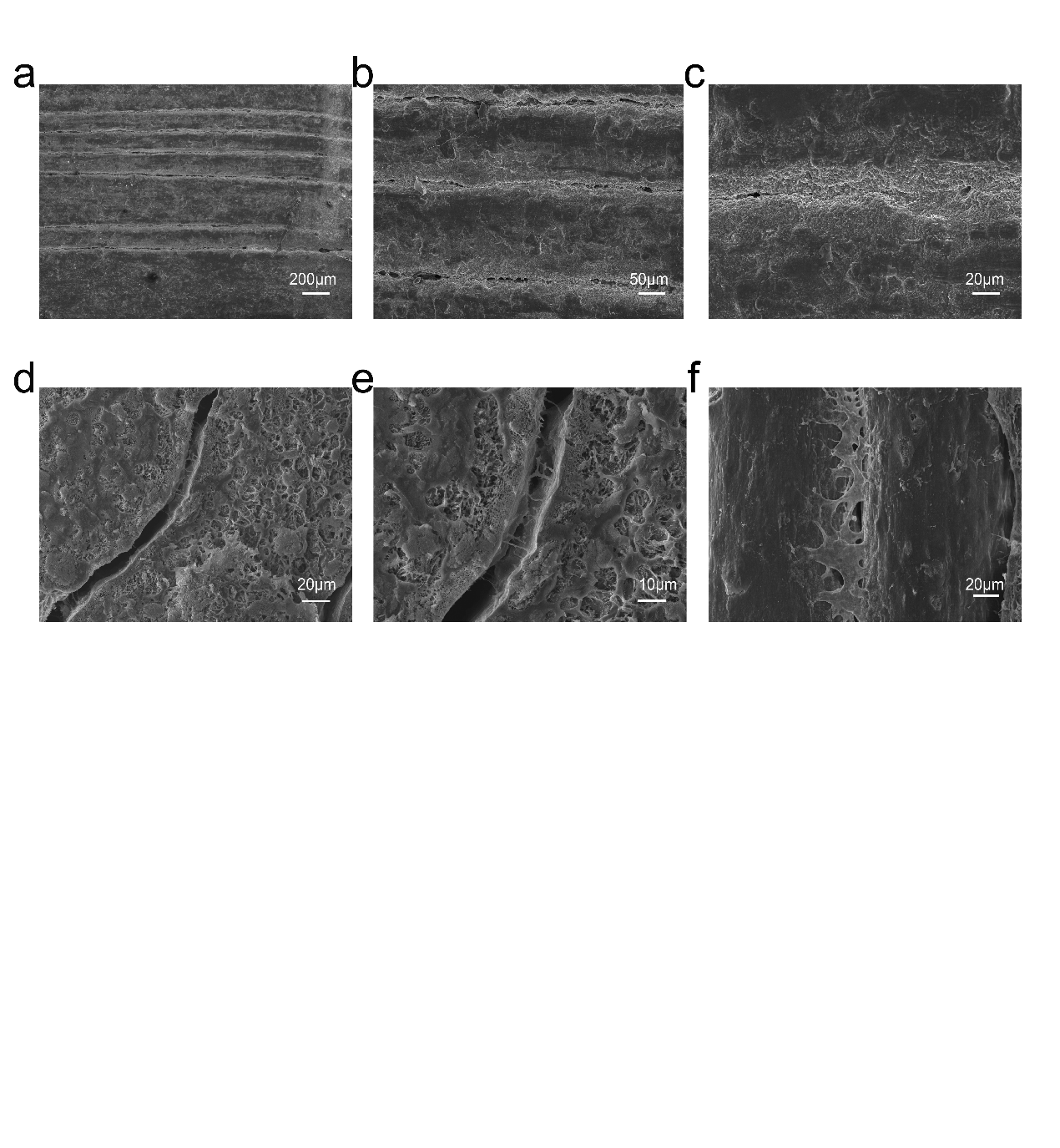


**Figure S6.** (a-f) SEM images of adjacent fiber filaments in BDHs and connections between them.


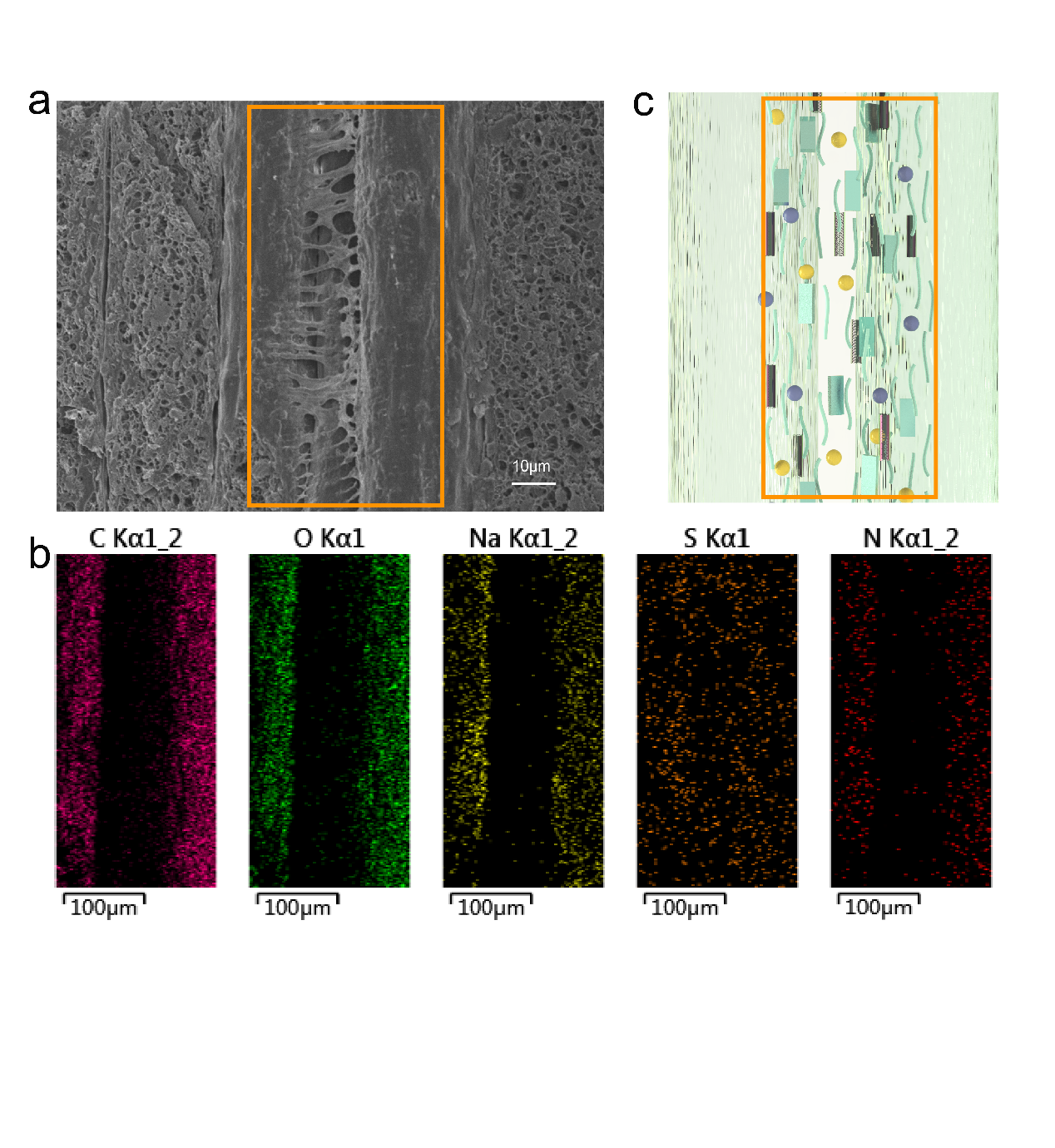


**Figure S7.** (a) SEM image of adjacent fiber filaments in BDHs and (b) corresponding EDS elemental mapping showing the distribution of a series of elements in the gap between adjacent fiber filaments. (c) The schematic illustration of the potential factor contributing to the formation of connections.


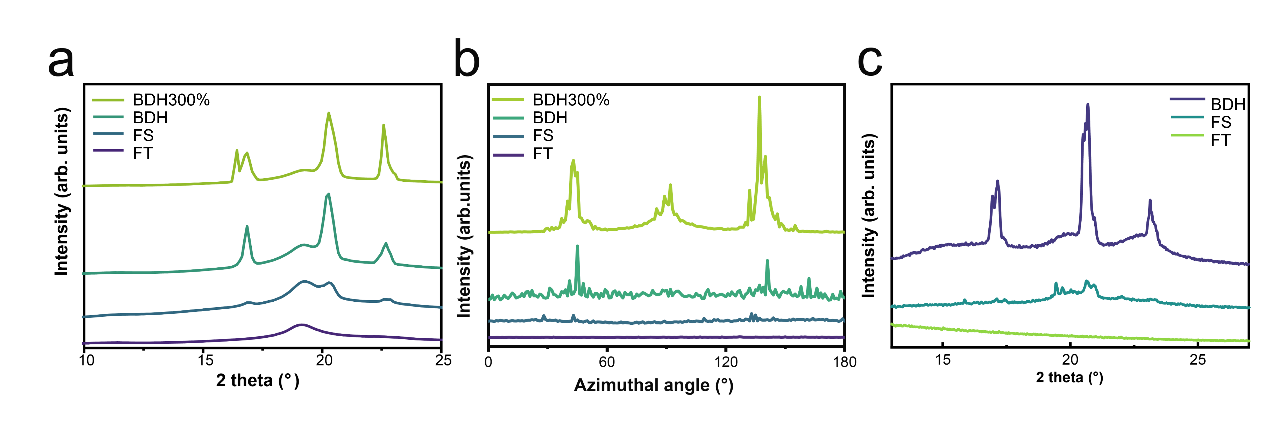


**Figure S8.** (a) WAXS profiles of the resulting hydrogels. (b) Scattering intensity I versus azimuthal angle θ curve of the resulting hydrogels. (c) XRD diffraction patterns of FT, FS and BD hydrogels, illustrating the phase composition and crystallinity.

**
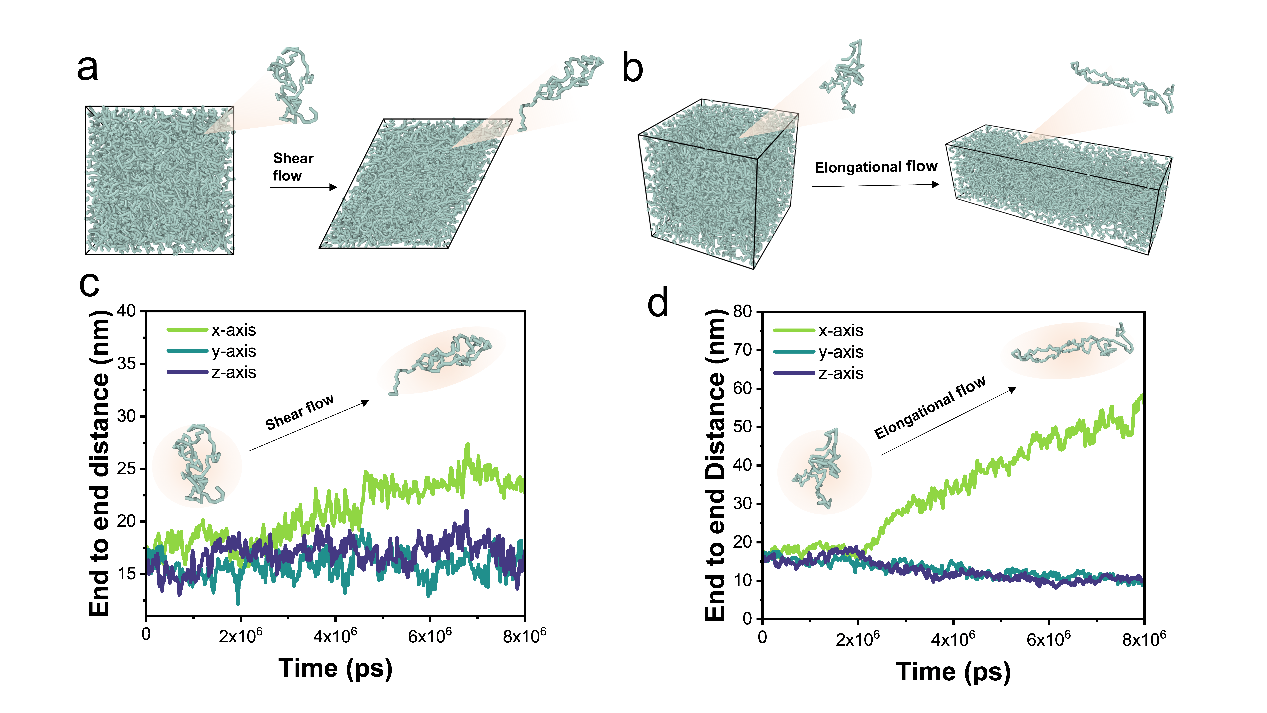
**

**Figure S9.** Molecular dynamics simulations of PVA chains during (a) extrusion and (b) stretching. Time evolution of the cartesian components of the end-to-end distance under (c) shear flow and (d) extensional flow conditions.


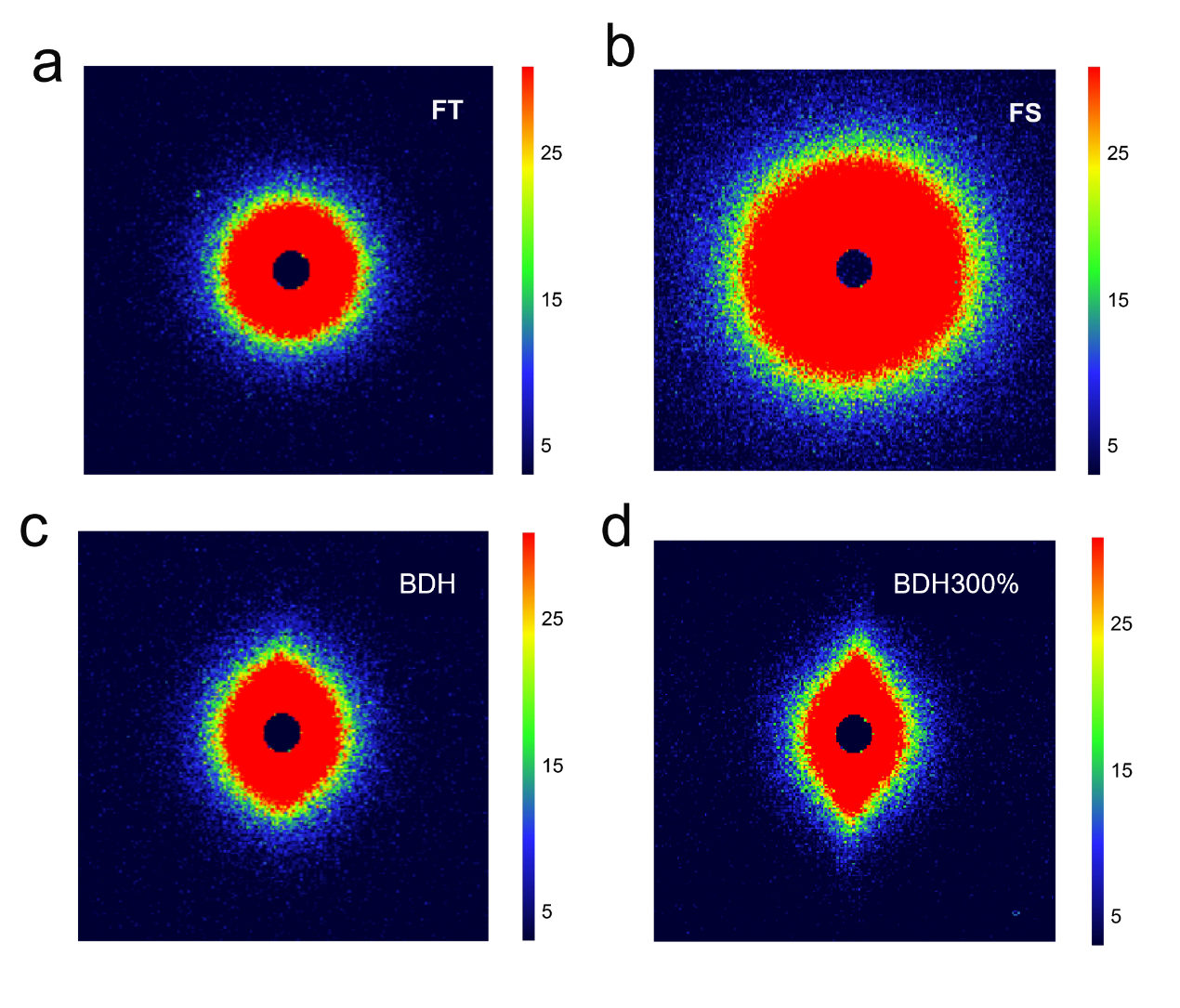


**Figure S10.** 2D SAXS patterns of (a) FT hydrogel, (b) FS hydrogel. (c) BDH and (d) BDH300%.


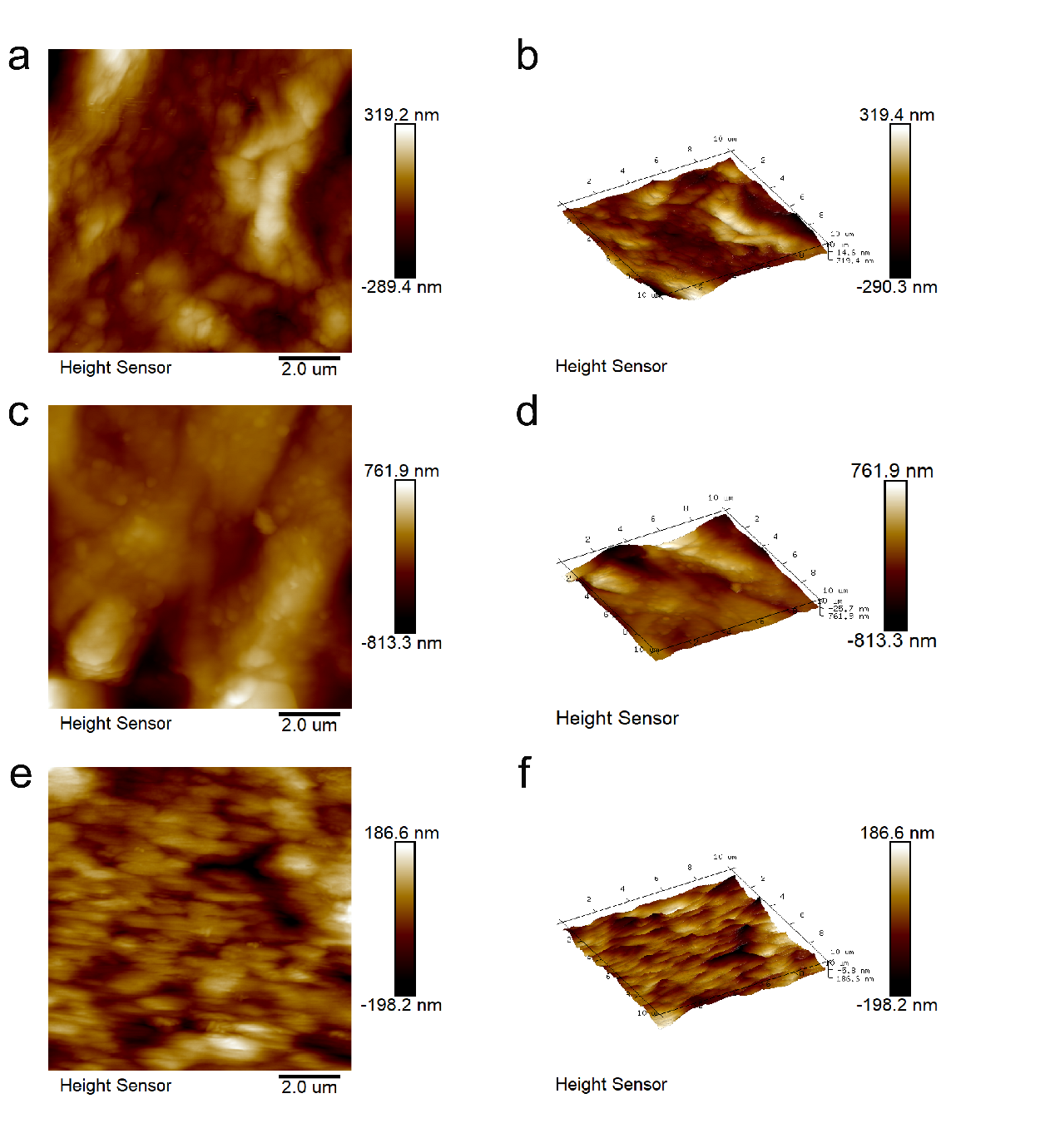


**Figure S11.** AFM images showing the microstructures of (a, b) FT, (c, d) FS hydrogels and (e, f) BDHs.


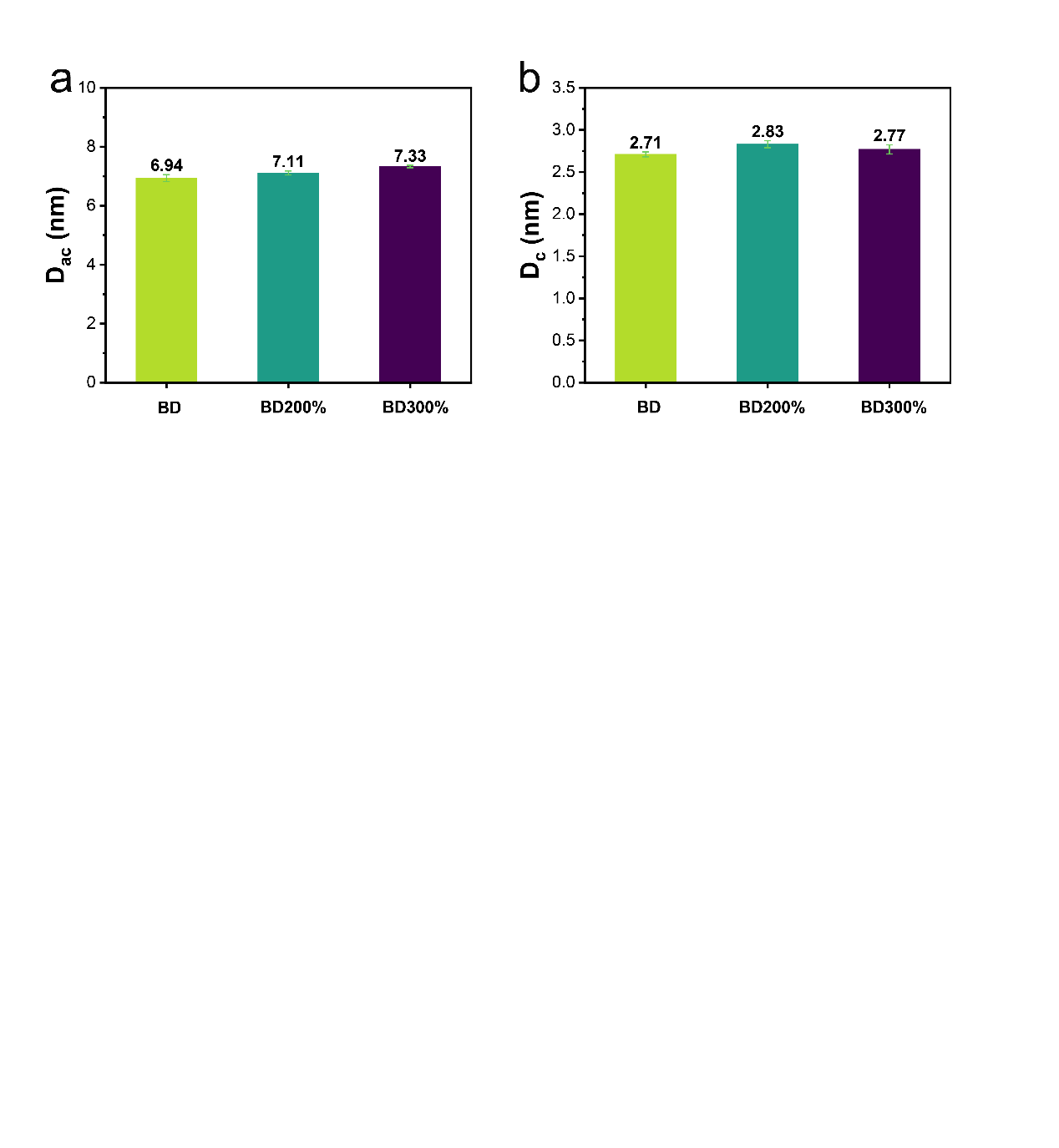


**Figure S12.** The changes of (a) the average distance between adjcent crystalline domains (D_ac_) and (b) the average size of crystalline domains (D_c_) during stretching.


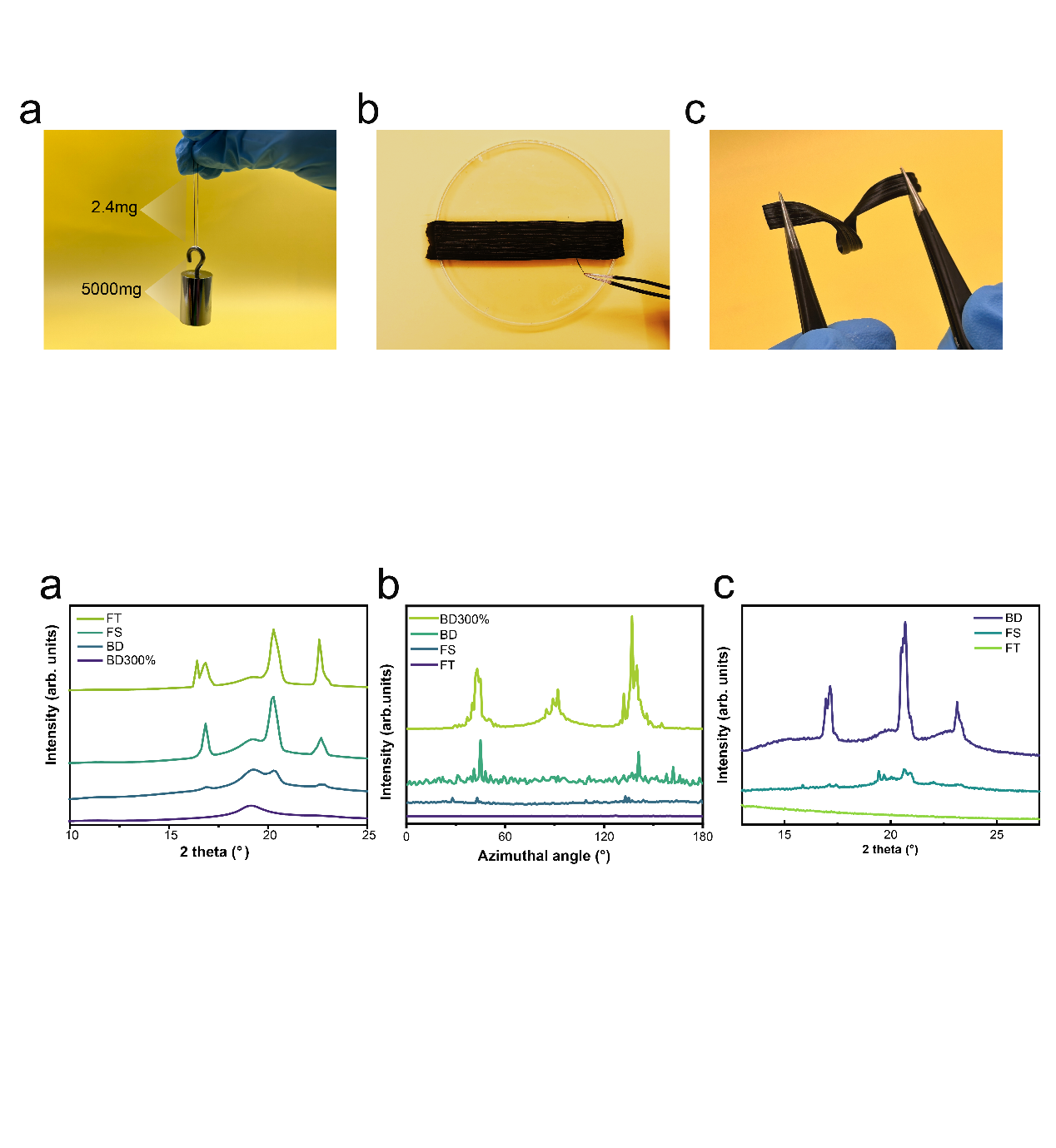


**Figure S13.** Typical photographs of (a) single fibrous gel under load, (b) BDH and conductive fibrous gel, (c) twisting deformation of BDH.


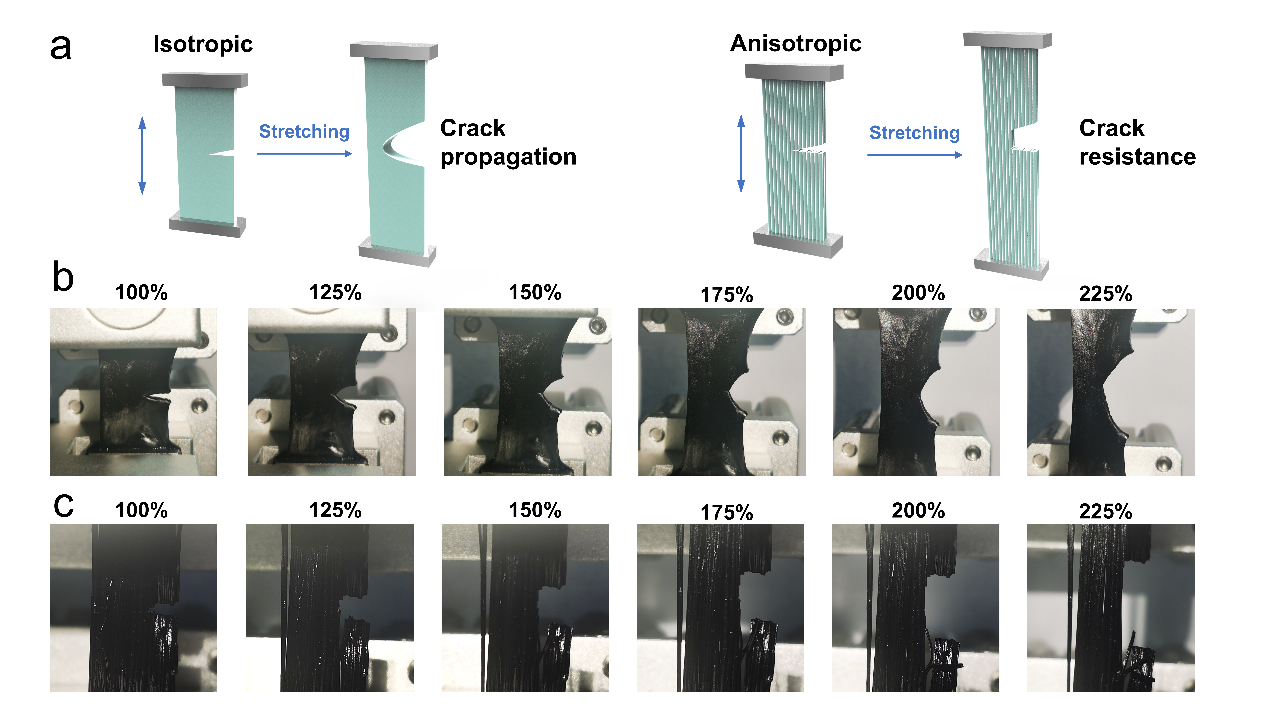


**Figure S14.** (a) Crack resistance of FS hydrogel and BDH under tensile stress. (b) Optical image of crack propagation in the isotropic hydrogel during stretching. (c) Optical image showing crack and resistance in the anisotropic hydrogel under the same condition.

**
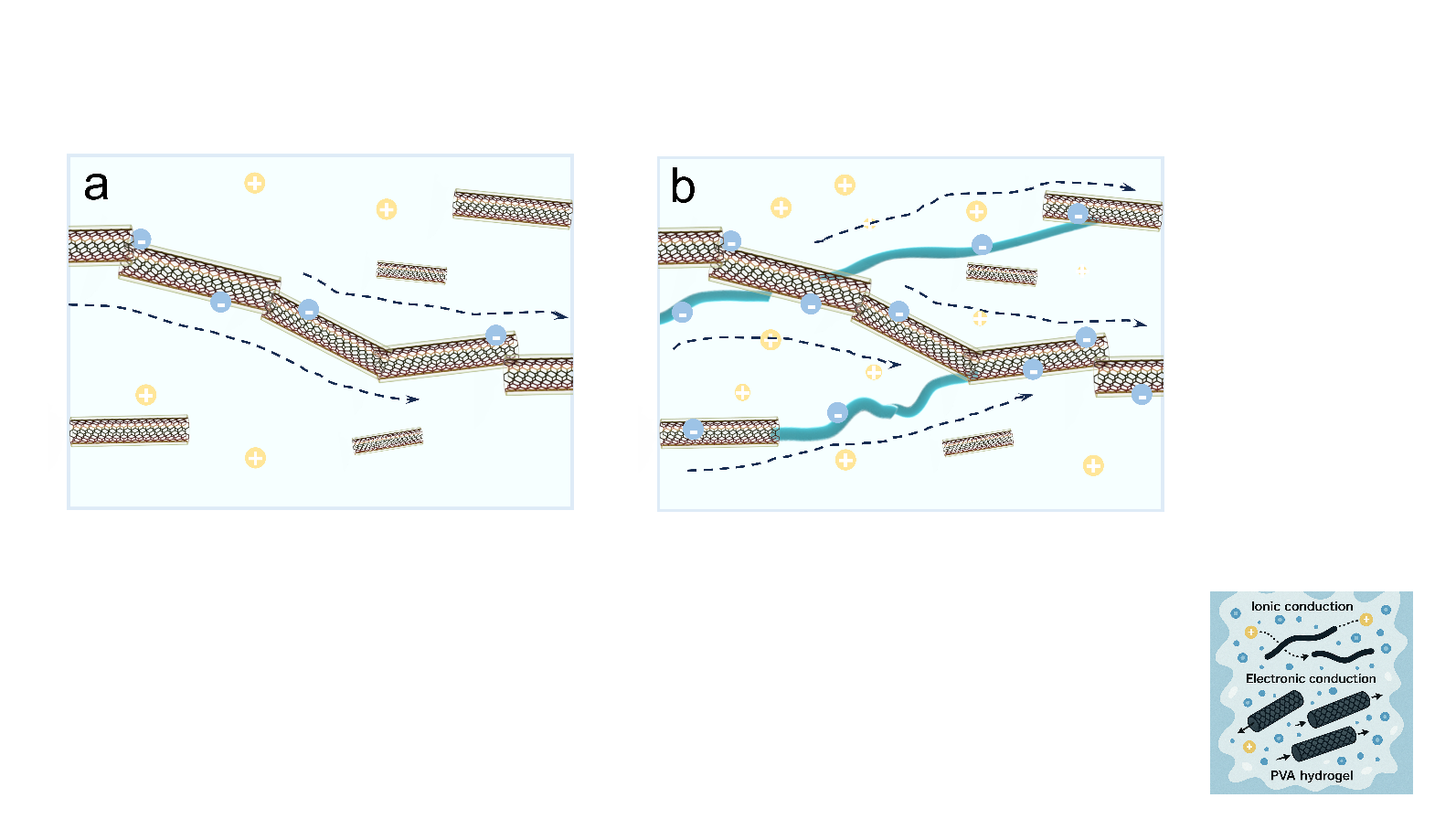
**

**Figure S15.** Electronic conduction mechanisms in the BDH system: (a) CNT-based conduction, and (b) enhanced conduction with the assistance of PEDOT:PSS.

**
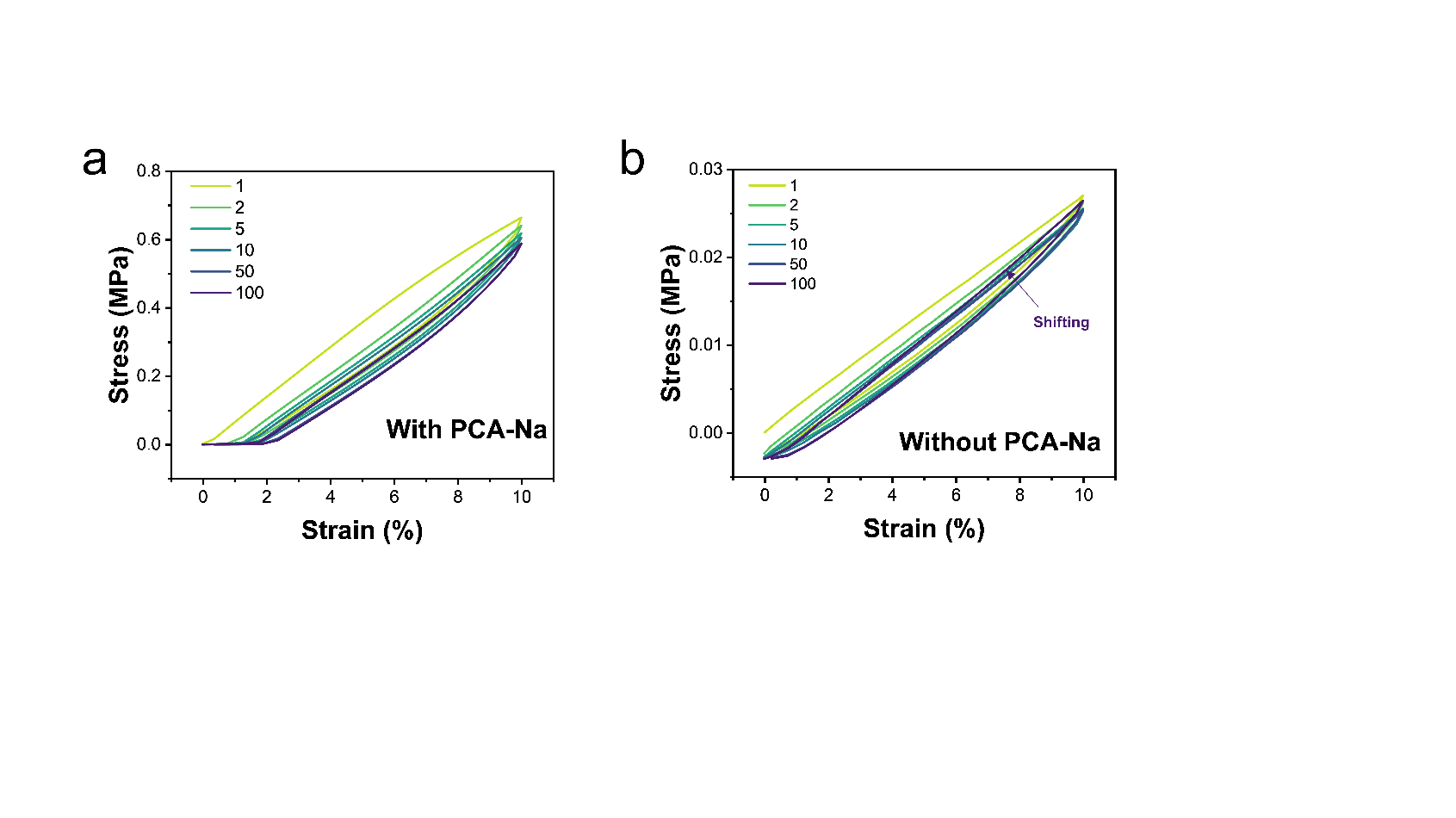
**

**Figure S16.** Stress–strain curves of BDHs (a) with and (b) without PCA-Na soaking under cyclic tensile loading. An increase in slope is observed near the 100th cycle due to water loss.

**
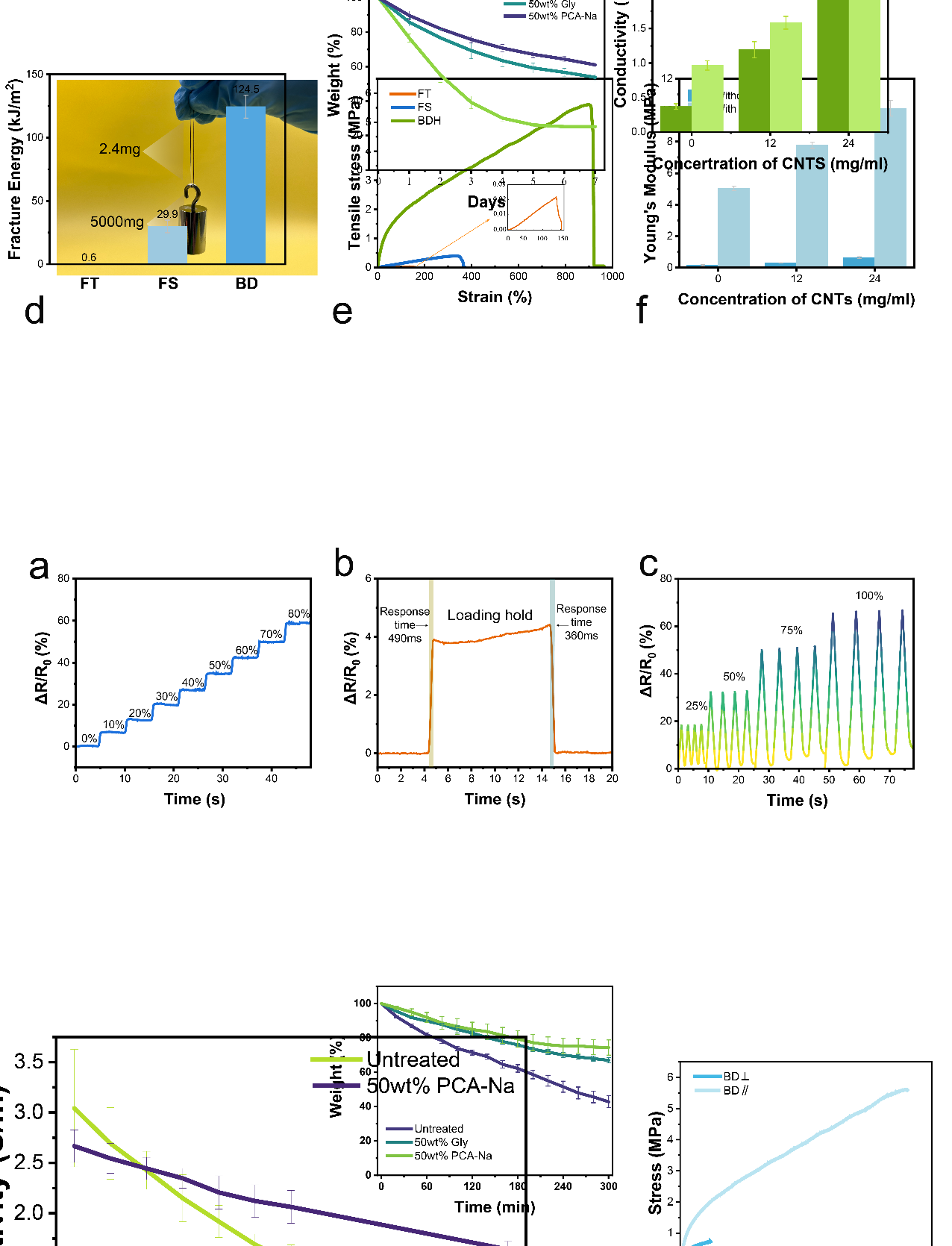
**

**Figure S17.** Sensing performance of BDHs under tensile strain. (a) Relative resistance variation of BDHs at strain. (b) Responsive time and recovery time of BDHs at 10% strain. (c) Resistance response of BDHs stretched to 25, 50, 75, and 100%.

**
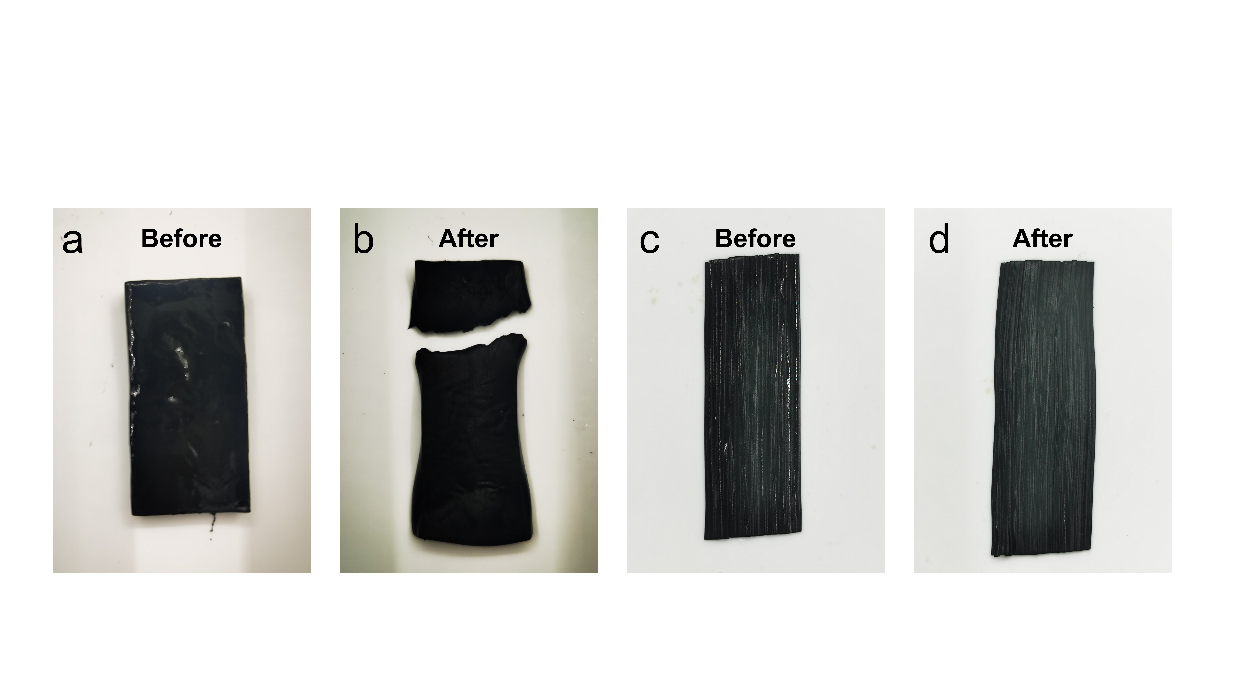
**

**Figure S18.** (a, b) FS hydrogels and (c, d) BDHs before and after being worn during intense physical activity by athletes, highlighting their performance under practical conditions.


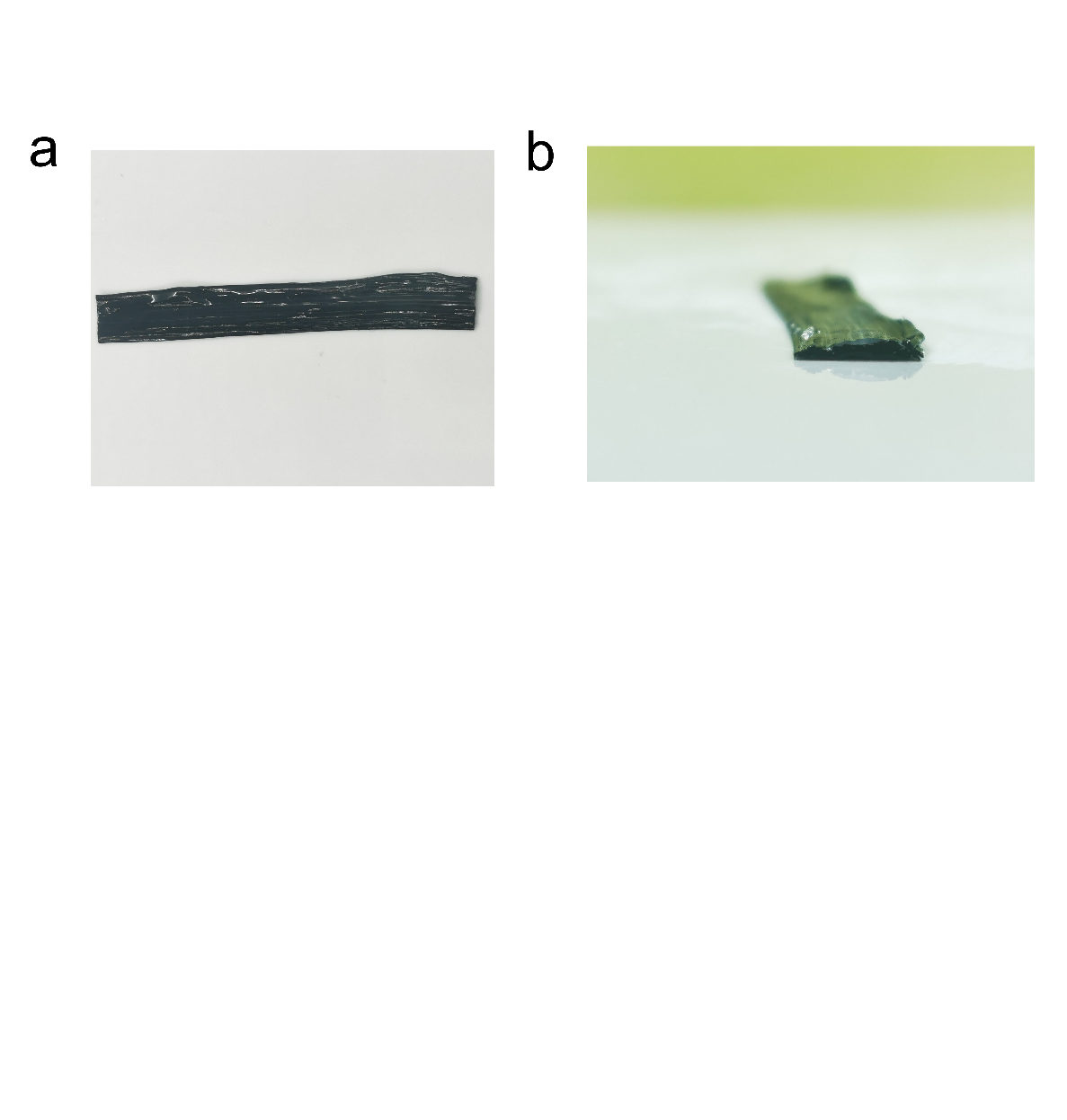


**Figure S19.** Optical images of BDHs encapsulated in silicone. (a) surface view and (b) cross-sectional view.


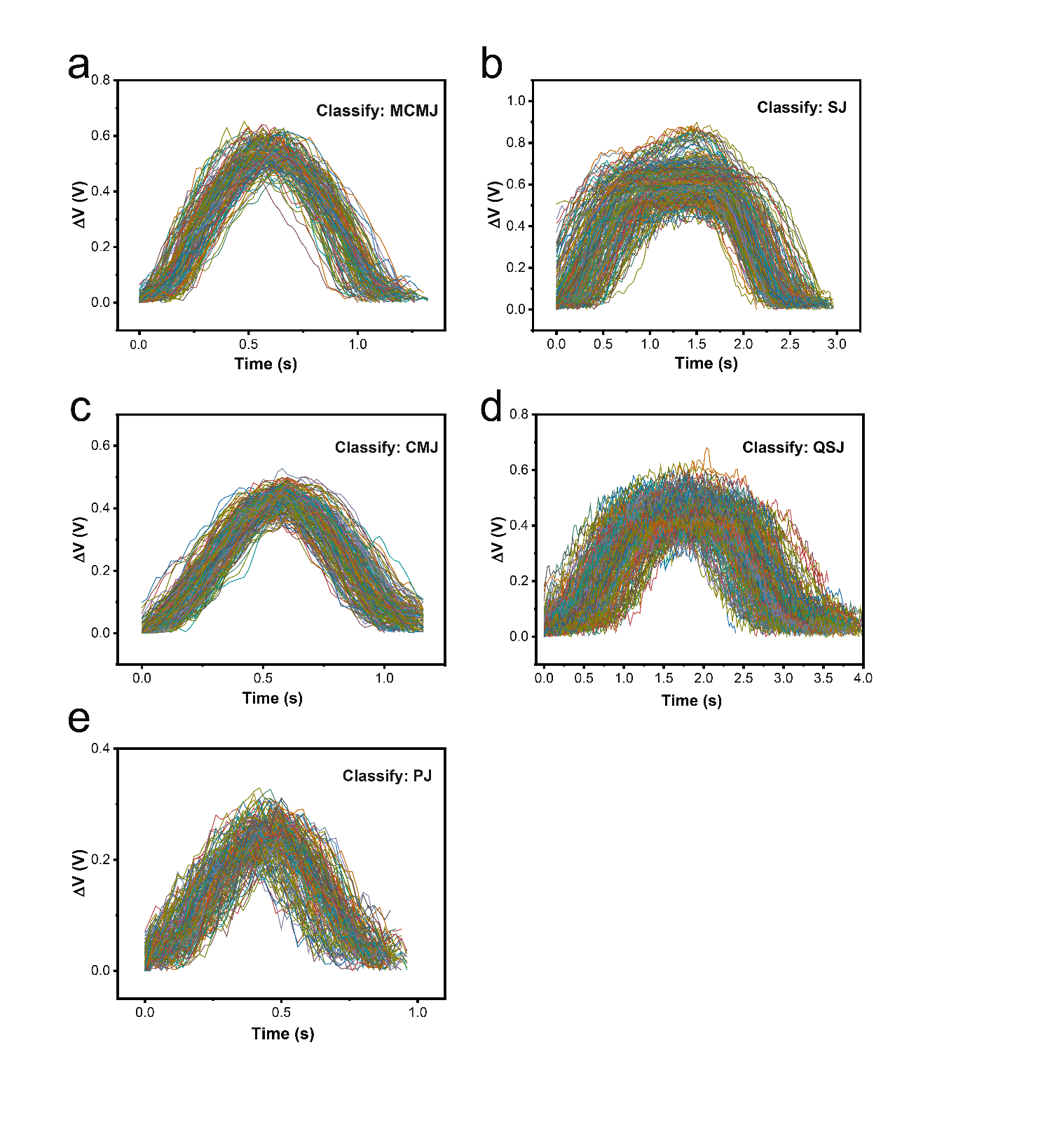


**Figure S20.** Original electrical signals recorded by BDHs during jumping movements: (a) Maximal effort countermovement jump, (b) Squat jump, (c) Countermovement jump, (d) Quanter squat jump and (e) Pogo jump groups.


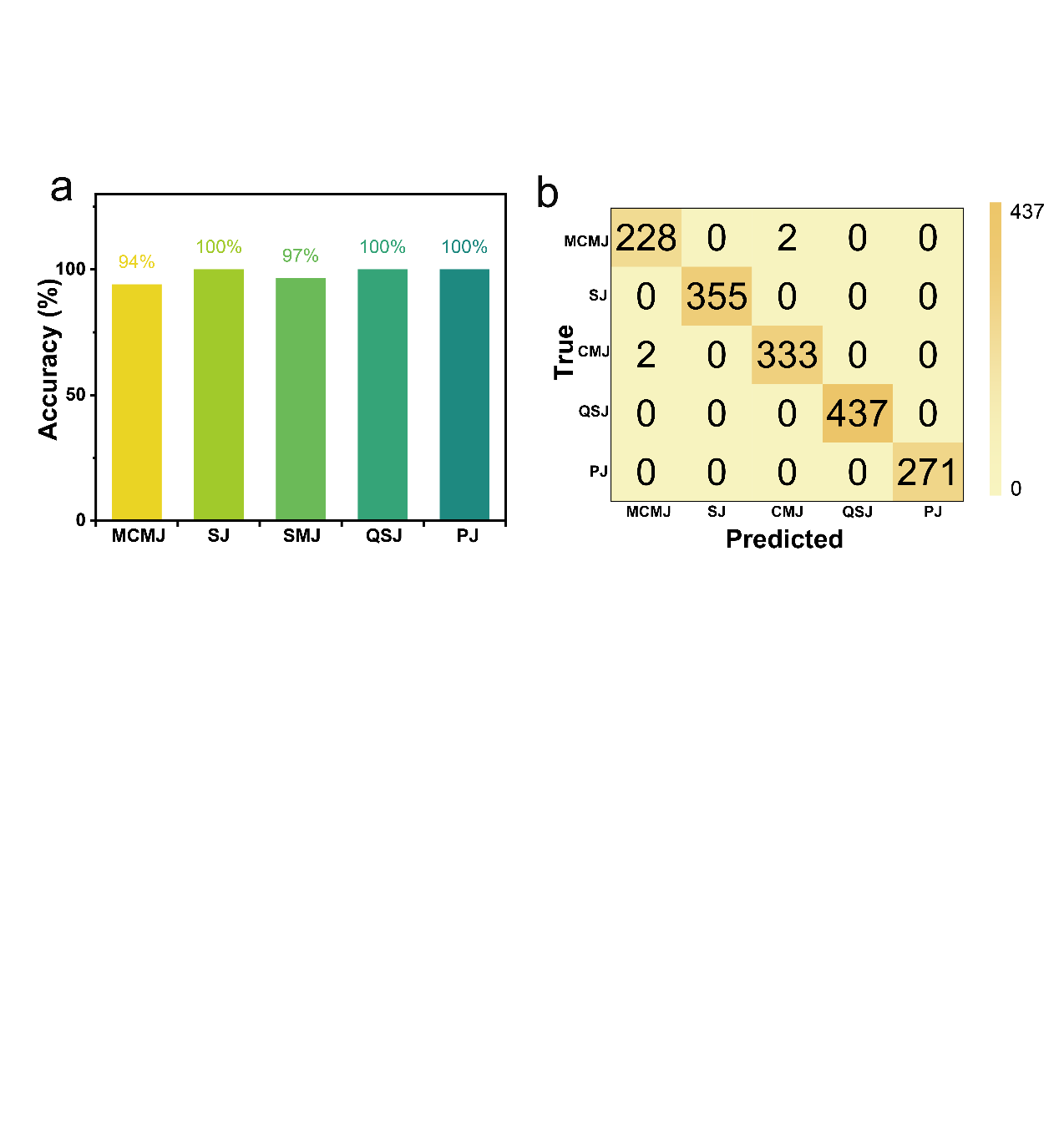


**Figure S21.** Performance evaluation. (a) Loss and accuracy function versus epochs in the training process. (b) Accuracy of each group for motion prediction. (c) Confusion matrix for the training group.
